# MicroRNA expression profiling in PBMCs of Indian water Buffalo (*Bubalus bubalis*) infected with Brucella and Johne’s disease

**DOI:** 10.1186/s41544-020-00049-y

**Published:** 2020-05-22

**Authors:** Jasdeep Singh, Jasdeep Kaur Dhanoa, Ratan K Choudhary, Amarjit Singh, Ram Saran Sethi, Simarjeet Kaur, Chandra Sekhar Mukhopadhyay

**Affiliations:** 1 College of Animal Biotechnology, Guru Angad Dev Veterinary and Animal Sciences University, Ludhiana, Punjab 141004 India; 2 Present address: University Institute of Biotechnology (UIBT), Chandigarh University, Mohali, Punjab 140413 India; 3 Department of Veterinary Pathology, Guru Angad Dev Veterinary and Animal Sciences University, Ludhiana, Punjab 141004 India; 4 Department of Animal Genetics and Breeding, Guru Angad Dev Veterinary and Animal Sciences University, Ludhiana, Punjab 141004 India

**Keywords:** microRNA, RNA-Seq, *Bubalus bubalis*, Brucellosis, Johne’s disease

## Abstract

**Background:**

MicroRNAs play key roles in host-pathogen-interactions and disease pathogenesis. Our aim was to characterize the differentially expressed miRNAs in the blood cells of diseased (Brucellosis-positive, Johne’s disease-positive) and healthy- water buffaloes. The pooled small-RNA samples of each group were sequenced on Ion Torrent Personal Genome Machine (PGM) sequencer and the data were analyzed for differential expression.

**Results:**

Here we identified 274 known miRNAs with bovine homologs and 36 novel mature-star miRNAs from the sequnces of small RNA libraries. Overall 195 miRNAs were common to all the three groups. Certain miRNAs such as bta-miR-21-5p, −26a, −29a/b, −30d − 103, − 140, − 150, − 191, − 374, − 1434-5p,-1260b, − 2484 and let-7 members were abundantly expressed in diseased groups. Bta-miR-1434-5p, − 188, −200c were up-regulated (> 1.5 folds) while bta-miR-27a-5p, −34b and -2285x were down-regulated (> 100 folds) in Brucellosis group. In Johne’s Disease group, only 3 miRNAs (bta-miR-1434-5p, − 2340 and − 2484) were up-regulated (> 1.5 folds). The functional classification of miRNA target genes into gene ontology (GO) terms indicated their involvement in innate immunity and cellular process of disease pathogenesis. Expression profile of four differentially expressed miRNAs (bta-miR-9-5p, − 677, − 331-3p and − 2440) and eight predicted target-genes were validated through reverse transcriptase qPCR.

**Conclusion:**

This study provides a valuable frame of reference for elucidation of regulatory roles of miRNAs associated with disease pathogenesis in water buffaloes as well as identification of miRNA biomarkers for disease diagnosis and treatment.

## Background

Circulating miRNAs have emerged as new disease-biomarkers in human cancer [1], and in animals [2–4]. The goals of this study were to identify PBMCs miRNAs profiles in Indian water buffalo associated with economically devastating diseases, namely, brucellosis and paratuberculosis (Johne’s disease or JD). Brucellosis an important zoonotic disease of livestock is caused by facultative, gram-negative bacteria of *Brucella* genus. This intracellular organism generally enters the host via the nasal and oral routes followed by invasion and proliferation within monocytic phagocytes [5]. It spreads via macrophages to the lymph nodes, spleen, liver, bone marrow, mammary glands, and reproductive organs. Another important disease that has been encountered in dairy animals is the paratuberculosis (Johne’s disease or JD) caused by *Mycobacterium avium* subspecies *paratuberculosis* (MAP). Calves mainly acquire the infection through oral route by uptake of MAP via colostrum, milk or feed contaminated with fecal matter [6] or via intrauterine route [7]. Following the invasion, the bacteria primarily resides in the mucosal tissues of gut and its associated lymph nodes and spreads to bloodstream, milk and other peripheral tissues [8]. MAP being an obligate intracellular pathogen resides in host macrophages and enhance its survival by inhibiting intracellular phagosomal activation and maturation [9, 10]. Thus, Brucellosis and JD, causes substantial economic losses to livestock worldwide and hence scientific research has been focused on their diagnosis, prevention, and control.

The currently available diagnostic tests of MAP infection, which are based on assays of IFN-γ and PCR, have the limitation of sensitivity and specificity, particularly in detecting early stage of infection [11, 12]. Therefore search for novel and alternative diagnostic biomarkers such as microRNAs for early stages of MAP infection would be beneficial.

The microRNAs (miRNAs) are short (~ 22 nt), non-coding RNAs that regulate post-transcriptional expression of mRNAs of at least one-third of known mammalian genes. These miniature RNAs play an instrumental role in immune regulation and disease pathogenesis [13–15]. Moreover, circulating miRNAs in the serum or plasma has also been reported. De-regulation of certain miRNAs indicates their utilization as potential biomarkers for various types of cardiovascular, nervous system diseases and cancer [16–18]. However, their possible association with the diseases in livestock species, particularly in buffalo, is the least studied.

A plethora of reports are available on immune and cytokine responses of Brucella infected peripheral blood mononuclear cells (PBMC) which have been exploited either for diagnosis or for vaccine development [19–22]. Similarly, recent studies on transcriptional profiling of PBMCs in cattle suffering from Johne’s disease have identified upregulated expression of genes (such as IL-5 & transcription factor GATA-3) promoting growth and differentiation of hematopoietic progenitor cells and T-lymphocytes genes as well as activation of neutrophils and macrophages. Further, infection with *M. paratuberculosis* is shown to be associated with upregulated expression of genes (Bad, CIDE-A, Fas, TNFRI) responsible for promoting apoptosis as well as genes (tissue inhibitors of matrix metalloproteinases, TIMP-1 and TIMP-2) having role in modeling extracellular matrix and tissue [23]. Additionally, natural infection of cattle with *M. avium paratuberculosis* (MAP) resulted in upregulated expression of the CD40 receptor and its ligand in PBMC [24].

Some immune-related miRNAs were up-regulated in various cells in response to intracellular pathogens, viz. mycobacterium infections [25, 26]. Further, some studies conducted in human have revealed significantly altered circulating miRNA profiles in serum of patients affected by tuberculosis than their respective healthy controls [27, 28]. Therefore, corroborating the possiblity that other intracellular mycobacterial and Gram-negative bacterial infections including Johne’s disease and Brucellosis of cattle may also alter the miRNA profiles of PBMCs. Very limited reports are available on the bubaline miRNA repertoire in context to disease. Only one such recent study characterized the serum and vaginal fluids miRNAs involved in Brucella infection in water buffaloes and implicated its use as potential disease prognostic biomarkers [29]. Recently our lab also identified isomiRs related to Brucellosis and Jhone’s disease in Indian water buffaloes and classified their predicted targets based on their biological and immune functions [30, 31].

In this study, we hypothesized that intracellular bacterial infections with MAP and Brucella causes significant changes in the miRNA profiles of bubaline PBMCs and profiling the repertoire of those miRNAs could help in identifying biomarker(s) for the diagnosis as well as disease resistance/susceptibility of animals. We performed next-generation sequencing of small-RNAs (sRNAs) extracted from the PBMCs of diseased (Johne’s disease and Brucella infected) and healthy buffaloes. The comparison of the catalogued-miRNAs between the diseased and healthy animals further provided a reference for future studies aimed to identify disease biomarkers and to modulate the expression of specific miRNAs for the disease therapeutics. The identified differentially expressed miRNAs and their predicted target genes were validated using real-time PCR.

## Materials and methods

### Animals

Diseased vis-a-vis healthy water buffaloes (*Bubalus bubalis*) included in the study were from the dairy farm of our institution i.e. Guru Anagad Dev Veterinary and animal Sciences University (GADVASU), Ludhiana as well as other dairy farms situated in Punjab, based on the history of occurrences of Johne’s disease and Brucellosis (abbreviated as “JD” and “Br”, respectively). The experimental animals were adult (3 years age) male or female buffaloes of Murrah (abbreviated as “Mu”) breed.

### Diagnosis of disease

Animal Disease Research Centre (ADRC), GADVASU performed the disease diagnosis (Brucellosis, and Johne’s disease). The Brucellosis-infected animals were diagnosed by the Rose Bengal plate test (RBPT), a rapid slide based agglutination test that detects antibodies against the smooth Brucella species particularly *B. abortus*, *B. melitensis* and *B. suis* [32]. The RBPT results were further confirmed by competitive ELISA (cELISA) using Brucella-Ab C-Elisa (Bovine) Kit (Svanova). The diagnosis of Johne’s disease was done by PCR using primers (IS900/150C: CCGCTAATTGAGAGATGCGATTGG; IS900/921: AATCAACTCC AGCAGCGCGGCCTCG) specifically amplifying 229 bp of target insertion sequence IS900 (i.e. present in multiple genomic copies in all *M. paratuberculosis* strains) [33]. The animals diagnosed positive for the diseases were segregated in different sheds of the respective herds. The experimental animals were divided into three diseased and one healthy control group:

- Brucellosis-positive Murrah buffaloes (Br-Mu; *n* = 2).
- Johne’s disease- positive Murrah buffaloes (JD-Mu; *n* = 5).
- Healthy Murrah buffaloes (Ctrl-Mu; *n* = 4) as a control group.

### Collection of blood samples and isolation of PBMCs

Peripharal blood samples (15 ml each) were aseptically collected from the jugular vein in an anticoagulant (0.5 M EDTA, 100 μl) containing collection tube (50 ml) and were kept at 4 °C using ice packs before further processing in the laboratory. The density-based gradient centrifugation method, using Hisep 1077 (HiMedia, Mumbai, India), was foolowed for PBMC isolation. The extracted PBMCs belonging to each of the specific experimental groups (i.e. biological replicates) were pooled into one sample. Since only two animal was tested positive for Brucellosis, about 30 ml of blood was collected from these animals in order to get approximately the same PBMCs count for all for the experimental groups. The three pooled samples of PBMCs were further processed separately for sRNA extraction and miRNA-sequencing [34].

### Small-RNA isolation and library preparation

The pooled PBMCs pellets were further processed for small-RNA extraction using *mir*Vana™ miRNA Isolation Kit (Ambion, Life Technologies, CA, USA). The steps included organic phase extraction and RNA immobilization on glass-fiber filters to yield enriched miRNA-fraction. The sRNA samples were quantified and tested for quality on Agilent’s Bioanalyzer (Agilent, CA, USA) before further processing. The electropherogram profile of each sRNA sample was obtained and the presence of degraded sRNA, as well as concentrations (pg/μl) of sRNA, was checked.

The extracted sRNA samples were transported in dry ice to GCC Biotech Pvt. Ltd., Kolkata for next-generation sequencing (NGS). The cDNA libraries were prepared using Ion Total RNA-Seq Kit v2 and assessed for quality on an Agilent 2100 Bioanalyzer with DNA 1000 Kit (Agilent Technologies, USA). Smear analysis was carried out by utilizing 2100 expert program (Agilent Technologies, CA, USA), to determine the size distribution of the amplified DNA. For template preparation, equal molar concentration (nM) each cDNA library were immobilized on Ion Sphere Particles for sequencing on Ion PGM™ Sequencer (Life Technologies, USA). The library workflow uses a proprietary technology to inhibits by-product synthesis and thus eliminating the need of the gel purification to select suitable size range. For better coverage and depth analysis, the samples were multiplexed on to two separate Ion Torrent chips (318 chip and 316 chip) .

### Bioinformatics analysis of miRNA-Seq data

The workflow for quantifying miRNA transcripts included pre-processing and quality assessment of reads, mapping of reads to bovine genome, and quantification of miRNA read counts by statistical analysis, differential mRNA expression and lastly target prediction and functional characterization.

#### Pre-processing of the raw reads

The three cDNA libraries were sequenced and the raw sequencing data were subjected to pre-processing steps to remove low quality reads containing too many missing nucleotides or reads of inappropriate length (i.e. either less than 18 nucleotides or more than 30 nucleotides). Clean reads were further obtained by trimming adaptor sequences and saved as fastq files which records the unique sequence and its count. Further quality control check of filtered reads was performed by K-mer analyisis.

#### Alignment to bovine genome and identification of mature as well as novel miRNAs

Since *Bubalus bubalis* complete genome was not sequenced, all the filtered sequences were mapped to the cattle reference genome (Btau_4.0 assembly), which is the most closely related species to buffalo. Moreover, sRNA sequencing data generally covers sequences of tRNAs, piRNAs, snRNAs and snoRNAs and repeat sequences. So to filter out such sequences, the quality-checked reads were then aligned to different libraries viz. Rfam database (http://rfam.xfam.org/), Repbase database (http://www.girinst.org/server/RepBase/), tRNA, hairpins and piRNA database (http://pirnabank.ibab.ac.in/about.html). To identify to known miRNAs and predict novel miRNAs, filtered reads were aligned to miRNA database i.e. miRBase Release 20 (http://www.mirbase.org/) and categorized with respect to the previously known miRNAs of *Bos taurus* species. For attributing imperfect Dicer processing of precursor miRNAs, certain short nucleotide extensions at 3′ and 5′ terminal were taken into account. Moreover, mutations and RNA editing sites were identified within the miRNAs by aligning the reads to pre-miRNA. Normalized read counts were calculated by taking the ratio of miRNA read counts times one million to the total number reads for each experimental group and then transformed to natural log scale:

$$ \log read\ count s={\log}_e\left(\frac{Read\ count\ of\ miRNA\ast 1000,000}{Total\ read\ count s\ matched\ in\ the\ sample}\right) $$

For the purpose of novel miRNA prediction, the unmapped read sequences were checked for RNA folding properties by using RNAfold programme in Vienna package and the reads aligning to parts of precursor miRNA, i.e. mature sequence, loop sequence and star sequence or miRNA* less expressed sequence, were extracted. The criteria adopted for further shortlisting such sequences as putative novel miRNA is that, it should be positioned on either arm of the folded secondary structure, have < 25 kcal/mol minimum free energy (MFE) and have conserved homologous sequences in other species.

#### Expression profiles

The Venn Diagrams online tool (http://bioinformatics.psb.ugent.be/webtools/Venn/) was used for graphical depiction of overlapping and uniquely expressed miRNAs the diseased and healthy groups viz. Br-Mu, JD-Mu and Ctrl-Mu, respectively. The miRNAs abundance data across the three groups, were were transformed to a logarithmic scale. The dispersion of expression profiles of the three experimental groups has been graphically presented by box-plots. The heatmaps were constructed using the R program’s WGCNA package to show the expression of all the miRNAs (a total of 262) identified in three samples [35]. The expression level of each miRNA in the respective groups is depicted in accordance to color scale bar where white-orange-green-red, represents increase in expression levels.

#### Prediction of miRNA targets and its functional characterization

For the purpose of predicting the putative mRNA targets of differentially expressed miRNAs we used combination of three online tools viz. TargetScan [36], miRDB [37, 38] and PicTar [39]. The top few hits of each tool were screened to select only those predicted targets having a role in innate immune response and disease pathogenicity. The significance (the probability that the predicted target is conserved) of the predicted target is obtained by P_CT_ (< 1) in TargetScan, highest target-score(s) for miRDB and highest PicTar score(s) for PicTar tools. The miRNA targets were further analysed based on the gene ontology, using online tool PANTHER Classification System (http://pantherdb.org/) and functionally categorized into different biological processes and molecular functions [40].

### Validation of miRNAs and target genes expression

Four differentially expressed miRNAs (2 miRNAs for each diseased vs. healthy groups), identified based on NGS fold-change, and a pair of predicted target genes for each of these four miRNAs (i.e. 8 target genes) were selected for experimental validation. The target genes were chosen based on the information available on their expression in PBMCs in humans (http://www.genecards.org/). These selected miRNA and miRNA-target genes were validated by qRT-PCR in an independent set of healthy and diseased buffaloes. The freshly isolated PBMCs from the blood of diseased and healthy buffaloes were individually processed using miRNeasy Minikit (Qiagen, Hilden, Germany) for total RNA that includes miRNA fraction.

#### Reverse transcription

For quantifying miRNA expression, cDNA was prepared from total RNA (1 μg for each sample) using the miScript II RT kit (Qiagen, Hilden, Germany). The details of SYBR-green primer sequences for miRNAs and miRNA target genes are listed in Tables 1 and 2, respectively.

**Table 1:** Sequence and reason of selection of the differentially expressed miRNAs used for validation

**SN**	**miRNA**	**miRNA primer name**	**Primer Sequence (5′-3′)**	**Comments on the specific miRNA**^**a**^
1	bta-miR-9-5p	BbuMir16-F	ucuuugguuaucuagcug	To compare Br-Mu with Ctrl-Mu
2	bta-miR-677	BbuMir19-F	cucacugaugagcagcuu	
3	bta-miR-331-3p	BbuMir18-F	gccccugggccuauccua	To compare JD-Mu with Ctrl-Mu
4	bta-miR-2440	BbuMir21-F	ugcagugaugagacccug	
5	Endogenous Control	BbuRNU6	acgcaaauucgugaagcguu	

^a^Br-Mu: *Brucella* infected buffaloes of Murrah breed

JD-Mu: *Johne’s Disease* infected buffaloes of Murrah breed

Ctrl-Mu: Healthy buffaloes of Murrah breed

**Table 2:** Detail of the designed primers used for qPCR validation of the selected miRNA-target genes vis-à-vis their functions

**SN**	**miRNA**	**Target** **Gene**	**Function of Target Gene**	**Target gene Primer Name &Sequence (5′-3′)**	**Tm (°C)**	**Ampli-con length**
1	bta-miR-9-5p	Y-Box Binding Protein 3 (YBX3)	Associated with binding of nucleic acid, activation of transcription factor, Sertoli-Sertoli cell junction related pathways	YBX3-F: ccgcaatgccggtgagattg	62	187
				YBX3-R: gtttggaccattggccgctt	62	
2	bta-miR-9-5p	Sorting Nexin 25 (SNX25)	Phosphatidylinositol binding and type I transforming growth factor beta receptor binding.	SNX25-F: aaatgcgccaaaacccgaca	62	185
				SNX25-R: gcatttgctcgccgttctct	62	
3	bta-miR-677	Nuclear Receptor Subfamily 3, Group C, Member 1 (NR3C1)	As transcription factor and also as regulator of transcription factor; **Mutations:** generalized glucocorticoid resistance	NR3C1-F: acctacgcagtgaaatgtcagact	62	162
				NR3C1-R: gtttctccatatttggcattgctgt	60	
4	bta-miR-677	Transmembrane 9 Superfamily Member 3 (TM9SF3)	Regulation of gene expression, morphogenesis, and differentiation, cell cycle progression.	TM9SF3-F: cgctatggtgtgtggcactg	62	170
				TM9SF3-R: gctgacctgacagatttcggc	62	
5	bta-miR-331-3p	Benzodiazepine Receptor (Peripheral) Associated Protein 1 (BZRAP1)	GPCR & downstream signaling of B Cell Receptor. **Association with diseases:** amelogenesis-imperfecta disease.	BZRAP1-F: acggctgtgctggagaactt	62	182
				BZRAP1-R: caggcgatctcggcagatgt	62	
6	bta-miR-331-3p	Cleavage and Polyadenylation Specific Factor 2, 100 kDa (CPSF2)	Gene expression and mRNA splicing pathways; RNA binding.	CPSF2-F: cgctgctgaaccaacgtcag	62	187
				CPSF2-R: cggtccaacaacaacaatccaaa	60	
7	bta-miR-2440	RAB39B, Member RAS Oncogene Family (RAB39B)	Encodes a member of the Rab family of proteins that are involved in vesicular trafficking. **Mutations:** X-linked mental retardation.	RAB39B-F: acacgtccagccctaccaaa	61	189
				RAB39B-R: aatagcgtctcgggctgacg	62	
8	bta-miR-2440	Ribosomal Modification Protein RimK-Like Family Member A (RIMKLA)	Metabolism pathways and Alanine, aspartate and glutamate metabolism; glutathione synthase activity and N-acetyl-L-aspartate-L-glutamate ligase activity	RIMKLA-F: ccttcgaccaggcatgcaac	62	175
				RIMKLA-R: tagacgctctccgcaactcc	62	

#### Real-time PCR detection of miRNAs and target genes

The 10-μL reaction setup for miRNA qRT-PCR included about 5 ng of diluted cDNA, 5 μL of 2X QuantiTect SYBR Green PCR Mix (Qiagen, Hilden, Germany), 1 μL of 10X miScript Universal primer and1 μL of 10X forward primer. The reaction was performed on ABI-7500-PCR System (Applied Biosciences, CA, USA) with the recommended cycling parameters i.e. initial heat activation (95 °C for 15 min), followed by a total of 40 cycles of denaturation (94 °C for 15 s), annealing (55 °C for 30 s) and extension (70 °C for 30 s). Each miRNA assay was run in a quadruplet (in two sets of experiments of duplicate biological and technical replicates). For relative miRNA quantification, the expression was normalized to bovine RNU6 endogenous control.

For target genes quantitation, was 20-μL reaction was setup for each gene assay (run in quadruplets) on Real-time PCR (7500 Applied Biosystems) with the same diluted cDNA (~ 5 ng) that was used for miRNA expression analysis, 2X QuantiTect SYBR Green PCR Mix (10 μL), 10 pmol forward & reverse primer (2 μL each) and RNase-free water. The cycle conditions were as follows: 15 min at 95 °C, followed by 40 cycles of 15 s at 94 °C, 40 s at 55 °C and 40 s at 70 °C. Bovine GAPDH and beta-actin were used as normalization control and a no-template control was also included.

#### Analysis of real-time qPCR data

The SDS software version 2.3 (Applied Biosystems, USA) was used for calculating threshold cycle (Ct) with baseline set at 0.2 threshold. Cycle threshold (Ct) values of miRNA or target genes were normalized to Ct values of endogenous controls as mentioned above for obtaining dCt values. Relative quantification (ΔΔCt) was performed by calibrating the dCt values of the diseased samples with respect to dCt values of control samples [41]. The fold-change with respect to the calibrator has been depicted graphically. The error bars indicate standard errors. The dCt values were subjected to one-way ANOVA to determine significant differences (*P* < 0.05, indicated by asterisks) between the healthy and diseased groups for the gene of interest (each miRNA and respective two target genes).

## Results

### Identification of miRNAs in bubaline PBMCs

We present the maiden report on in vivo study to identify and catalog the miRNA repertoire in water buffaloes, associated with Brucellosis and Johne’s disease. The three sRNA sequence raw data-files (in fastq format) have been submitted to NCBI-SRA (SRR3382673, SRR3382604, and SRR3383406).

The overview of miRNA sequencing data inclucing distribution of total initial reads, reads that mapped to miRbase, the number of known miRNAs identified, and number of novel miRNAs (i.e. miRNA sequences not reported in miRBase) is presented in tabular form (Table 3). A total of 274 miRNAs were identified across all the groups that matched to previously known miRNAs in bovine genome and additionally 36 novel mature* miRNAs (that originate from the less expressed arm of respective pre-miRNA) were identified. The list of identified mature miRNAs, its sequence and position as well as sequence their pre-miRNA; are provided in Supplementary files 1 and 2.

**Table 3:** Number of filtered reads number of reads matched to miRbase vis-à-vis total known and novel mature-star miRNAs identified in the four experimental samples

**SN**	**Group Name**	**Total Initial Reads**	**Reads matched to known mature miRNAs**	**Mature miRNAs**	Novel mature* miRNAs
**1**	**Br-Mu**	2,676,675	89,577	258	25
**2**	**JD-Mu**	2,288,179	153,334	228	23
**3**	**Ctrl-Mu**	198,993	94,798	232	12

Br-Mu: Brucellosis positive Murrah buffalo

JD-Mu: Johne’s Disease positive Murrah buffalo

Ctrl-Mu: Healthy buffaloes (Control group) of Murrah breed mature* miRNAs: mature miRNAs that originate from alternative or less expressed strand of precursor miRNA hairpin structure

**Fig. 1:**
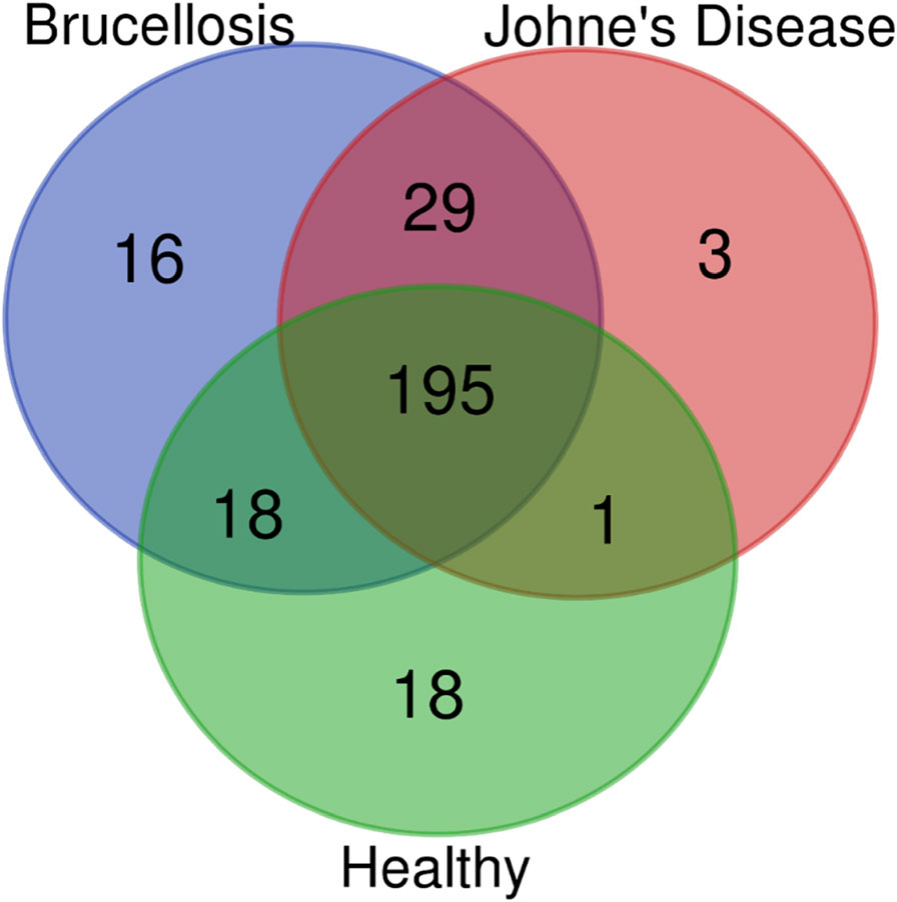
Venn diagrams representing common and unique miRNAs across the three groups a) Brucellosis-positive buffaloes b) Johne’s disease-positive buffaloes c) Healthy buffaloesprepared using an online tool (http://bioinformatics.psb.ugent.be/webtools/Venn)

Biocomputational analyses of the small RNA sequencing cataloged 258, 228 and 232 miRNAs in the BrMu, JDMu, and the control CtrlMu experimental groups, respectively (Supplementary file 4). It is evident from the Venn diagram (Fig. 1) that 195 miRNAs were common to all three groups. Some miRNAs (16, 3 and 18, respectively) were unique to each of the groups (Brucellosis, Johne’s Disease and Healthy, respectively) (Fig. 1). The identification of uniquely expressed miRNAs specific to particular group suggests association with disease resistance or susceptibility.

### Differentially expressed miRNAs

**Fig. 2:**
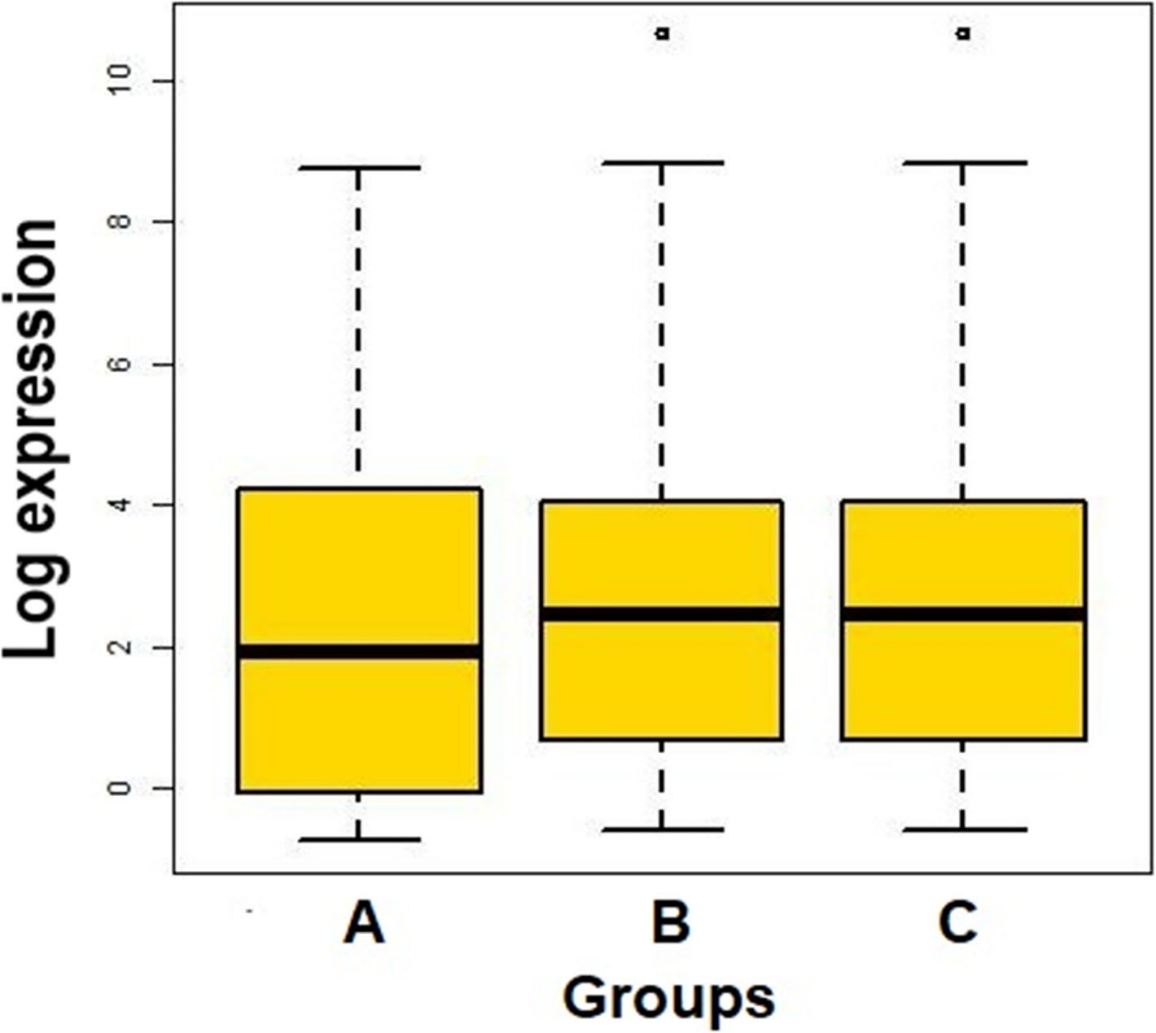
Box-plot showing an abundance of known miRNAs across diseased and healthy control samples. The overall abundance of 275 known miRNAs expressed as log-transformed FPKM values distributed across the three groups **a**) Brucellosis-positive buffaloes **b**) Johne’s disease-positive buffaloes **c**) Healthy buffaloes; shown as a box plot. The central line inside the boxes indicate median values, box width indicates 25 and 75% quartile ranges around the median, the termini indicate the 2.5 and 97.5 percentiles of the data, and the dots represent outliers

**Fig. 3:**
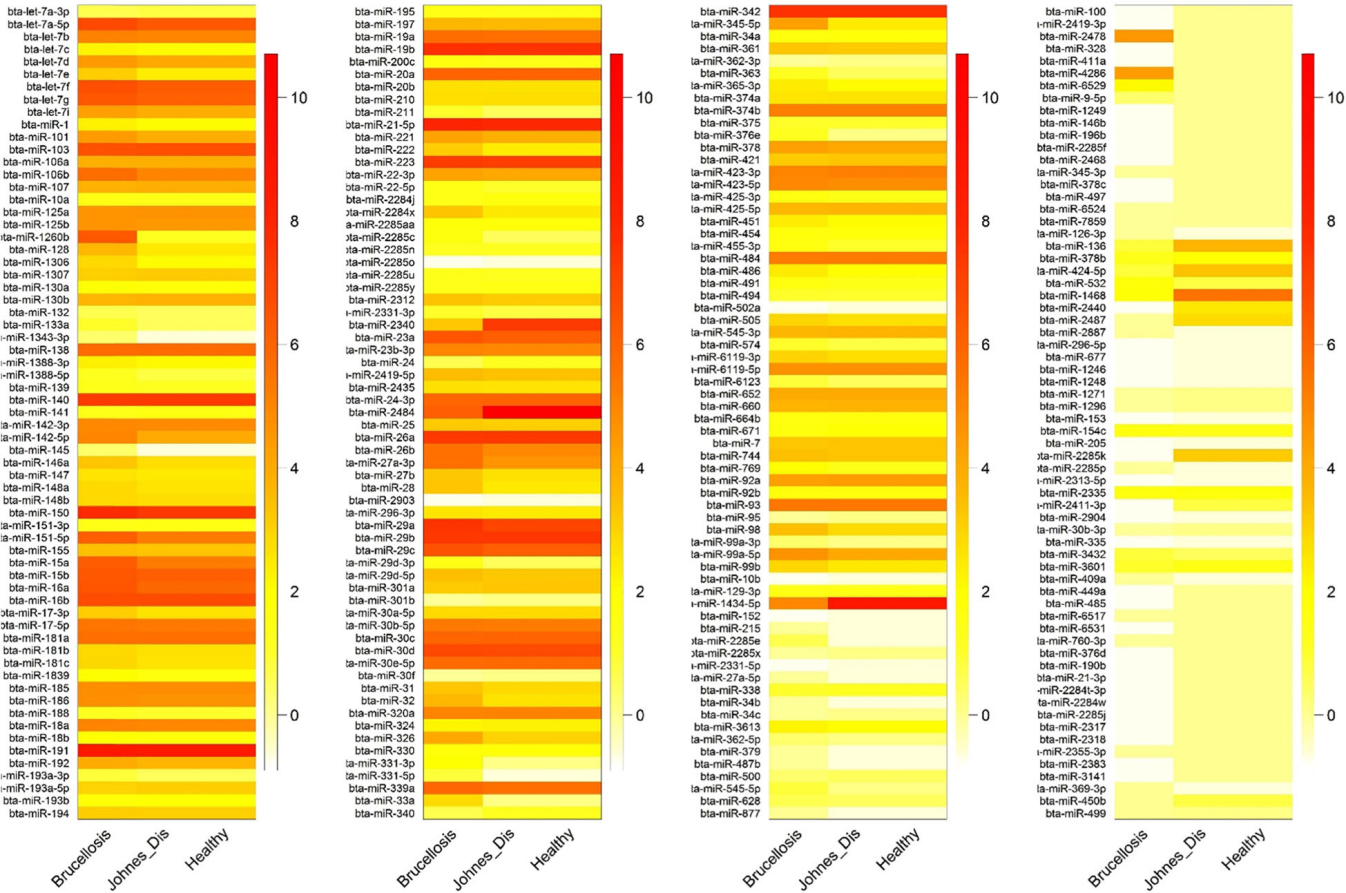
Heatmap of known miRNA expression across the diseased and control samples. The log expression levels of the known miRNAs (i.e. having taurine homolog) across the three groups viz. Brucellosis-positive buffaloes, Johne’s disease-positive buffaloes and Healthy buffaloes; are depicted by the heatmap. The color scale bar (white-orange-red) indicate increasing expression levels (low-medium-high)

Group-wise distribution of the overall abundance of known miRNAs (log-transformed FPKM values) is shown as a box plot (Fig. 2). The mean abundance of the miRNAs was higher in the JD-Mu and control group than Br-Mu group. The log expression levels of the known miRNAs (i.e. having taurine homolog) across the three groups are depicted by the heatmap (Fig. 3). In Br-Mu group, bta-miR-191 and -21-5p had highest expression. While, bta-miR − 2484 and − 1434-5p were most abundantly expressed in the case of JD-Mu group. Likewise in Ctrl-Mu groups, miRNAs viz., bta-miR − 150, −19b, −let-7a-5p, − 223, −let-7f were abundantly expressed along with high expression of bta-miR-191 and -21-5p. Most of the identified miRNAs in the three groups had moderate to low expression levels.

The normalizied expression level of miRNAs were compared in the disease-groups and the healthy control group to identify differentially expressed miRNAs (Supplementary file 3). Based on the criteria of fold change of 1.5 or greater, only one miRNA i.e. bta-miR-1434-5p showed upregulation in Brucella infected (Br-Mu) buffaloes. Whereas several miRNAs in this group were downregulated (fold change > 2), including bta-miR-27a-5p, −2285x and -34b, which exhibited more than 100 folds decreased expression as compared to healthy water buffaloes of control group (Ctrl-Mu).

Further, 3 miRNAs viz. bta-miR-1434-5p, − 2340 and − 2484 in Johne’s diseased (JD-Mu) buffaloes group, were expressed at higher levels (> 1.5 folds) in comparison to Ctrl-Mu, of which bta-miR-1434-5p showed highest expression of 3.12 folds. Moreover, in comparison to control group (Ctrl-Mu) of animals, many miRNAs were significantly down-regulated (> 2 fold change). It was interesting to note that 11 miRNAs within this group were downregulated from 30 to 52 times, with bta-miR-331-3p exhibiting utmost downregulation (51.5 folds) .

### Functional categorization of miRNA targets

**Fig. 4:**
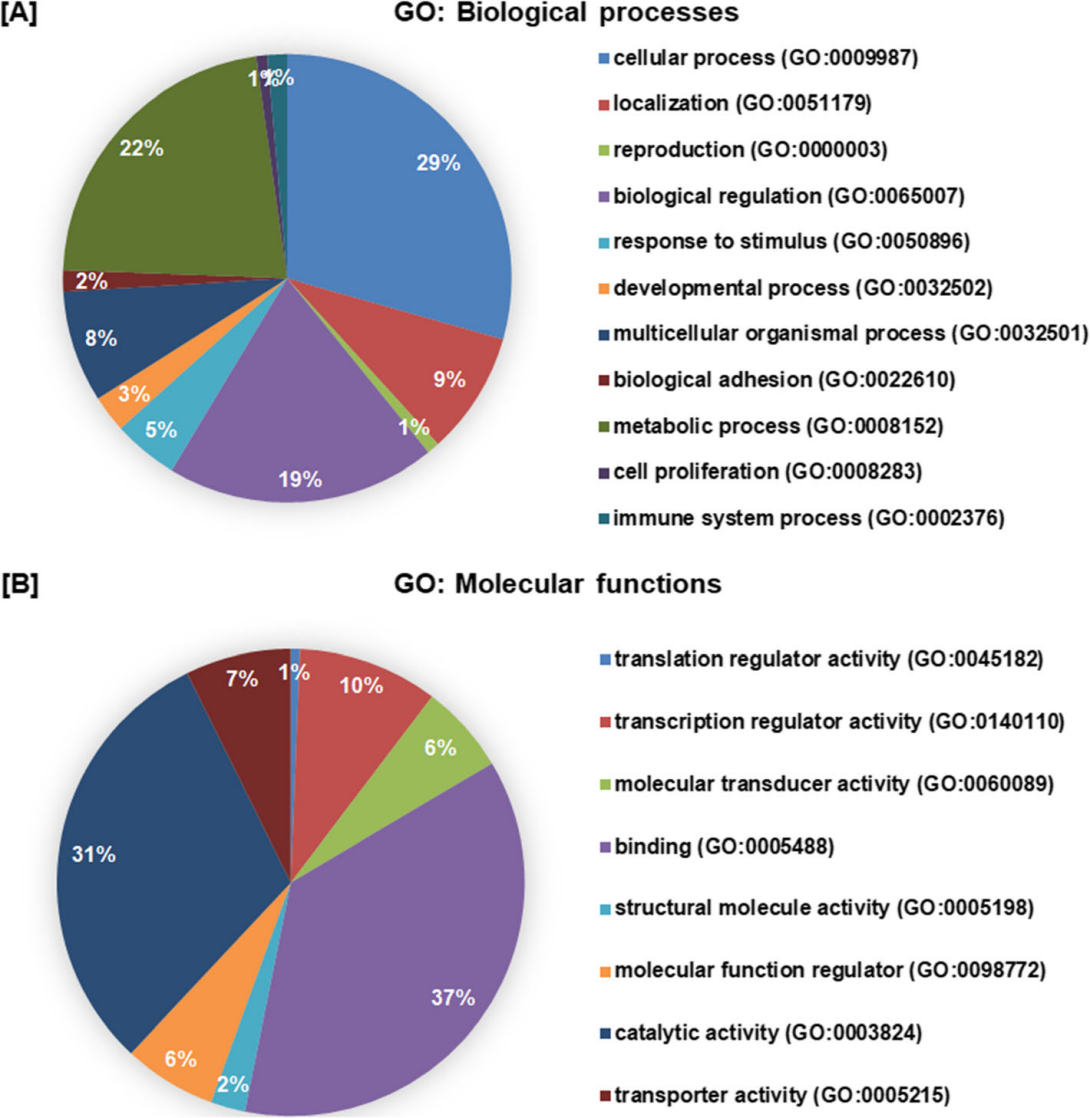
Gene Ontology (GO) based classification of the miRNA targets. A total of 937 predicted miRNA-target genes were functionally classified into various categories biological processes (**a**) and molecular functions (**b**) using PANTHER Classification System (http://pantherdb.org/)

In order to further infer the role of differentially and uniquely expressed miRNAs in relation to disease, 937 gene targets were predicted by three online tools used in this study. The predicted targets were broadly classifed into biological processes and molecular functions (Fig. 4a and b) based on gene ontology (GO). The 1134 biological process hits includes 11 different pathway categories such as cellular processes (GO:0009987)- 29%, metabolic processes (GO:0008152)- 22%, biological regulation processes (GO:0065007)- 19%, adhesion processes, response to stimulus, cell proliferation and immune responses etc. Further, 794 hits of molecular functions were divided into 8 pathway categories including of binding function (GO:0005488)- 37% and catalytic function (GO:0003824)- 31%, among others.

### qPCR validation of selected differentially expressed miRNAs

The real-time expression of the four selected miRNAs viz. bta-miR-9-5p, bta-miR-677, bta-miR-331-3p, and bta-miR-2440 were analyzed (normalized against endogenous control BbuRNU6). There was ~ 0.4 fold expression i.e. 2.5 times down-regulation of bta-miR-9-5p and ~ 8 fold increase in the expression of bta-miR-677, in Br-Mu samples as compared to the healthy control (Ctrl-Mu). Further, bta-mir-331-3p and bta-mir-2440 showed 0.47 and 0.053-fold expression i.e. 2.12 and 18.87 times down-regulation, respectively, in JD-Mu as compared to the healthy control (Ctrl-Mu).

### qPCR validation of target genes

Two target genes (that are expressed in blood cells) for each of the above-selected four miRNAs were also selected to compare the expression of the target genes with the respective targeting miRNAs. Normalization of the Ct values of the target genes was done separately with two endogenous controls: beta-actin and GAPDH. It is evident that the fold-change in the expression of target genes was higher with respect to beta-actin than that of GAPDH as an endogenous control. The bta-miR-9-5p (down-regulated 2.5 times in Br-Mu as compared to control) miRNA target genes viz. YBX3 and SNX25, showed up-regulated expression (2.09 and 5.14 folds) in Br-Mu than in control with beta-actin as endogenous. The up-regulated expression of SNX25 i.e. 5.14 folds, in Br-Mu was significantly different (*P* value = 0.0001) than in the control group. These target genes, YBX3 and SNX25, showed 0.63 and 1.84 fold change with respect to GAPDH, respectively. Similarly when comparing the Br-Mu versus control, one of the target genes of miRNA bta-miR-677 (up-regulated ~ 8 folds) i.e. TM9SF3 was found to be down-regulated 1.12 times with respect to beta-actin. While it showed significant (at *P* value = 0.028) downregulation of 5.31 folds) in Br-Mu than in control, with respect to GAPDH. While it’s second predicted target gene i.e. NR3C1 expression was found to be significantly (P value = 0.004) up-regulated by 4.93 folds with respect to beta-actin. Moreover, it showed slightup-regulation (1.09 folds) in Br-Mu than in control, with respect to GAPDH.

In JD-Mu versus control group, the target genes viz. BZRAP1 and CPSF2; RAB39B and RIMKLA; showed down-regulated expression in JD-Mu with respect to control, even when both the miRNAs (bta-miR-331-3p and bta-miR-2440, respectively) were down-regulated in JD-Mu. Although the downregulation of both predicted targets of bta-miR-331-3p i.e. BZRAP1 and CPSF2 in JD-Mu were significantly different at P value = 0.021 and 0.002 respectively, than in the control group.

**Fig. 5:**
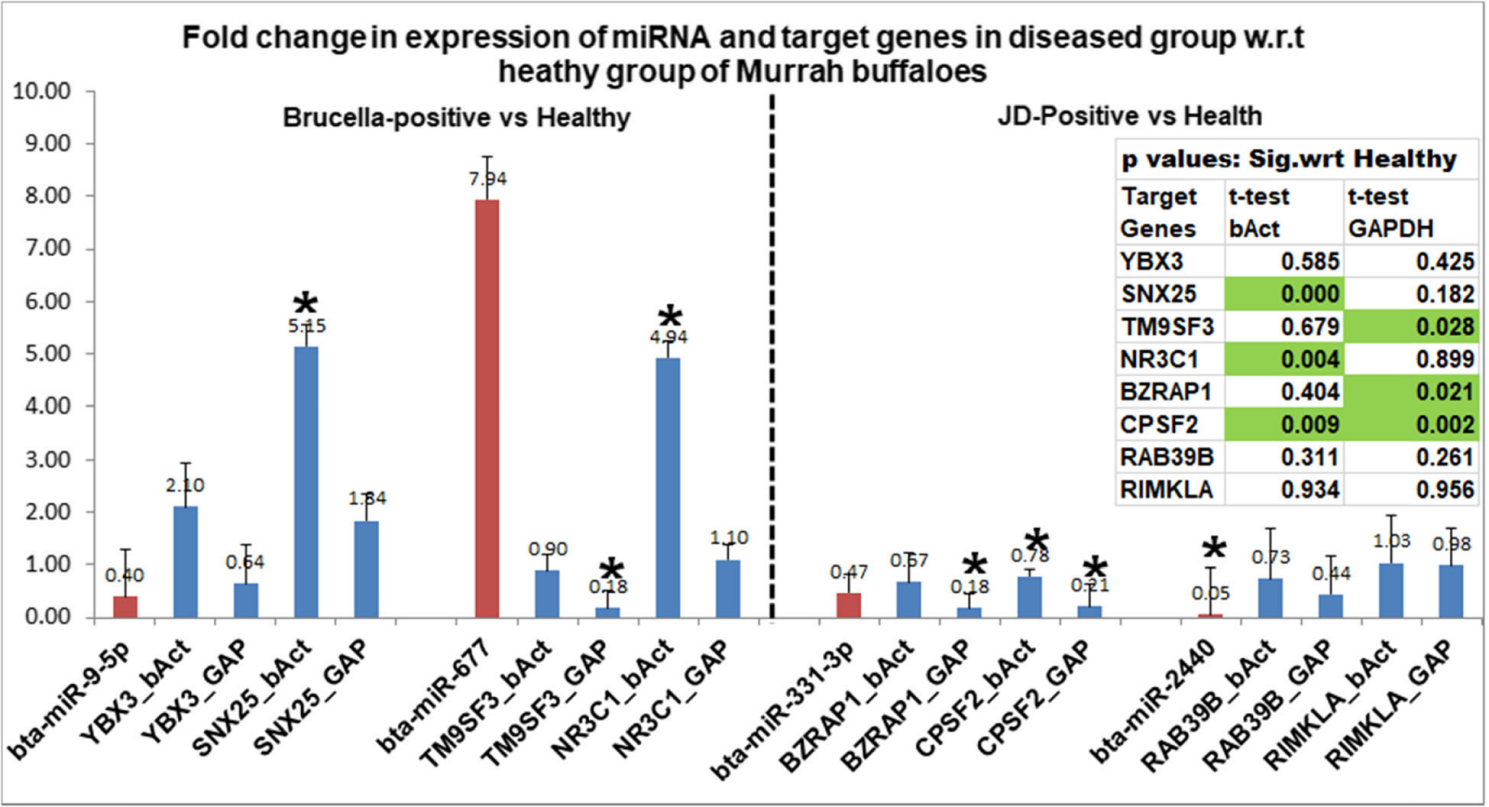
Relative expression profiles of selected miRNAs and the corresponding target genes. The real-time expression of the four selected miRNAs viz. bta-miR-9-5p, bta-miR-677, bta-miR-331-3p, and bta-miR-2440 were analyzed and normalized against endogenous control BbuRNU6. Two target genes for each of the above-selected four miRNAs, i.e. 8 target genes, were analyzed and gene expression was normalized against two endogenous controls: GAPDH and beta-actin and represented as fold change in diseased vs. control group. Asterisks indicate statistically significant differences (*p < 0.05) in diseased vs. healthy control group. The table indicating *P* values is included in the figure

The graph (Fig. 5) showed the expression of miRNAs normalized to RNU6 endogenous control along with their respective predicted target genes, normalized to beta-actin and GAPDH endogenous controls. The bars with the asterisk are significantly different at *P* < 0.05 in the diseased group than in the healthy control group.

## Discussion

Micro-RNAs (miRNAs) play a central role in regulating gene expression. Aberrant miRNA expression has been associated with human disease and pathological conditions, including cancer, neurodegenerative and cardiovascular diseases [42–44]. However, until now, a handful of studies have investigated the potential role of circulating miRNAs in infectious bacterial diseases [45–51]. Further, finding circulating miRNA signatures is more fascinating choice, for instance sampling is minimal invasive and packaging of extracellular miRNAs within vesicles or their association with proteins makes them less susceptible to degradation [13, 52].

Our current work is the first-ever report on the cataloguing of differentially regulated miRNAs in PBMCs associated with Brucellosis and Johne’s disease in Indian water buffaloes. Untill now, very limited literature is available regrading miRNA expression in response to bacterial infections of cattle. In one such study encompassing miRNA association with bovine mastitis, Lawless and co-workers identified 21 differentially expressed miRNAs by applying next-generation sequencing (NGS) approach in culture of bovine mammary epithelial cells challenged with *Streptococcus uberis* [53]. Jin and colleagues (2014) identified 231 known and 113 novel miRNAs associated with *S. aureus* or *E. coli* infections in vitro culture of bovine mammary epithelial cells (MAC-T cells). Five of these miRNAs viz. bta-mir-21-5p, − 22-3p, −27b, − 184 and let-7f had abundance greater than 50% [51]. Among the top expressed miRNAs described above, we also found high expression of bta-miR-21-5p and bta-let-7f in the PBMCs of Brucella infected water buffaloes.

Recently bovine TB associated miRNA profile was identified from the PBMC (stimulated with PPD-B) of unvaccinated and BCG-vaccinated cattle challenged with *M. bovis* [54, 55]. A core list of 88 known circulating miRNA including five (miR-22-3p, miR-92a, miR-191, miR-423-5p, and miR-486) most abundantly expressed miRNAs have been identified in the serum of cattle infected by Johne’s disease [56]. The researchers further compared the miRNA expression before and after 6 months of infection and recorded 2 folds upregulation of bta-miR-205, and 2 folds downregulation of bta-miR-432 post infection.

The results reported herein suggest that most of miRNAs including bta-miR-374a were downregulated in bubaline PBMCs upon infection with *M. paratuberculosis*, which is in agreement with the report on human by Sharbati and co-workers [55]. However, few miRNAs (bta-miR-191, bta-miR-1434-5p, bta-miR-2484 and bta-miR-2340) exhibited increased expression in the PBMCs of *M. paratuberculosis* infected buffaloes. Out of these, bta-miR-191 was highly expressed in this group which corroborated by previous report in *M. paratuberculosis* infected cattle that described bta-miR-191 as most abundantly expressed miRNA [56].

We also identified and catalog the differentially expressed miRNAs, in PBMCs, related to Brucellosis disease in Indian water buffaloes. The results demonstrated modulation in miRNAs expression, 8 of which (viz. bta-miR-188, −200c, − 375, − 132, − 1343-3p − 365-3p, − 6123 and − 1434-5p) were up-regulated and more than 7 including bta-miR-9-5p, −27a-5p, −30f, −34b, −99a-3p, −2285x, and − 6524 were down-regulated in response to *Brucella* infection. The down-regulation was more than 10 folds. Interestingly as of now, only a single previous study indicated altered miRNA expression in the context of *Brucella melitensis* infection. The results demonstrate involvement of putative targets of dysregulated miRNAs (miR-92a, − 93, −181b − 1981 and let-7b) in various immune responses including cell death and autophagy [57]. Recently our lab explored the differentially expressed miRNAs in PBMCs culture, obtained from blood of Indian water buffaloes, challenged with ligands of toll like receptors viz. LPS, poly I:C, and CpG ODN to mimic the microbial infections and identified 160 mature and 130 novel miRNAs [58].

Microarrays are the one of the most extensively used method for studing transcriptional expression profiling. But, as microarrays are solution hybridization based assays, there is high variation in signal to noise ratio and less specificity. Hence, real-time PCR based techniques have been developed to detect precursor and mature miRNAs. Since miRNAs are present in minute quantity in serum and plasma, real-time PCR has been recognized as a promising technique for the detection and validation of blood circulating miRNAs as biomarkers [59]. Here in the present study, we used SYBR Green based qRT-PCR to the validate the NGS miRNA expression data. The same differential expression pattern (up or down-regulation) of miRNAs as observed by NGS were also seen by qPCR. For example, the bta-miR-9-5p and − 331-3p that were observed to be down-regulated (~ 12 and ~ 51 folds, respectively) in Br-Mu and JD-Mu, respectively, as compared to control; showed down-regulation (2.5 and 2.12 folds, respectively) by the qPCR with respect to RNU6 endogenous control. The miRNA bta-miR-677 that showed unique expression in Br-Mu group by NGS, was observed to be expressed highly (~ 8 folds) in the respective group as compared to control by reverse transcriptase qPCR.

In the present study, a total of 8 predicted target genes for miRNA target expression were selected using three different tools based on their function in innate immunity and disease pathogenesis. Out of these, 3 predicted target genes viz. YBX3, SNX25, TM9SF3, followed the common negative regulatory function of miRNAs where up-regulated expression of miRNA in Brucella positive group resulted in the downregulation of target gene expression or vice versa. However, recent findings suggest that miRNAs can also promote the target gene expression in association with microRNP complexes [60, 61]. An example of such a remarkable association was observed herein study as well where NR3C1 was found to be upregulated in the Brucella positive group having up-regulated bta-miR-677 expression. Likewise, in JD positive group, the down-regulated expression of target genes (BZRAP1 and CPSF2; RAB39B and RIMKLA) was observed even when the miRNAs (bta-miR-331-3p and bta-miR-331-3p, respectively) were downregulated.

## Conclusion

In conclusion, our study unveiled first data on miRNA expression profile of healthy and diseased and water buffaloes associated with susceptibility of Johne’s disease and Brucellosis. The study is likely to contribute significantly to understanding the role of miRNAs in the pathogenesis of bacterial infection as well as the identification of miRNA biomarkers for disease diagnosis and treatment.

## Supplementary information

**Additional file 1.**

**Additional file 2.**

**Additional file 3.**

**Additional file 4.**

## Data Availability

All relevant data are within the paper and its Supporting files. The raw data file of miRNA-seq (in Fastq format) analysis has been published in the NCBI-SRA database and is available without any restriction for public use: SRR3382673 (Brucellosis-positive Murrah buffaloes), SRR3382604 (Johne’s disease- positive Murrah buffaloes) and SRR3383406 (Healthy Murrah buffaloes).

## Abbreviations

miRNAs: microRNAs
nt: nucleotide
PBMC: Peripheral blood mononuclear cells
sRNAs: small-RNAs
GO: Gene ontology
JD: Johne’s disease
TB: Tuberculosis
Br: Brucellosis
Mu: Murrah breed
Ctrl: Control group
MAP: *Mycobacterium avium* subspecies *paratuberculosis*
ADRC: Animal Disease Research Centre
RBPT: Rose Bengal plate test
NGS: Next-generation sequencing
Panther: The Protein Analysis Through Evolutionary Relationships
BMEs: bovine mammary epithelial cells
TLR: Toll-like receptors
