# Supplementary material for: MicroRNA expression profiling in PBMCs of Indian water Buffalo (Bubalus bubalis) infected with Brucella and Johne’s disease
Source: ExRNA. 2020 May 22;2(1):8. doi: 10.1186/s41544-020-00049-y (PMC7242893; doi:10.1186/s41544-020-00049-y)
Supplement: Supplementary file 1 — Additional file 1. [file 41544_2020_49_MOESM1_ESM.doc]

**Supplementary file 1. List of the known miRNAs (homologous to reported taurine miRNAs) identified in the bubaline PBMCs from diseased vis-à-vis healthy control animals**

| **SN** | **microRNA** | **Mature miRNA sequence**  **(5’-3’)** | **Pre-microRNA sequence (5’-3’)** | **Position** |
| --- | --- | --- | --- | --- |
| 1 | bta-let-7b | ugagguaguagguugugugguu | cggggUGAGGUAGUAGGUUGUGUGGUUucaggguagugauguugcccccucggaagauaacuauacaaccuacugccuucc | 6 to 27 |
| 2 | bta-let-7c | ugagguaguagguuguaugguu | gcauccggguUGAGGUAGUAGGUUGUAUGGUUuagaguuacacccugggaguuaacuguacaaccuucuagcuuuccuuggagc | 11 to 32 |
| 3 | bta-let-7d | agagguaguagguugcauaguu | ccuaggaAGAGGUAGUAGGUUGCAUAGUUuucgggcagggauuuugcccacaaggagguaacuauacgaccugcugccuuucuuagg | 8 to 29 |
| 4 | bta-let-7e | ugagguaggagguuguauagu | cccgggcUGAGGUAGGAGGUUGUAUAGUugaggaggacacccaaggagaucacuauacggccuccuagcuuuccccagg | 8 to 28 |
| 5 | bta-let-7g | ugagguaguaguuuguacaguu | aggcUGAGGUAGUAGUUUGUACAGUUugagggucuaugauaccacccgguacaggagauaacuguacaggccacugccuugcc | 5 to 26 |
| 6 | bta-let-7i | ugagguaguaguuugugcuguu | cuggcUGAGGUAGUAGUUUGUGCUGUUggucggguugugacauugcccgcuguggagauaacugcgcaagcuacugccuugcua | 6 to 27 |
| 7 | bta-let-7a-5p | ugagguaguagguuguauaguu | ugggaUGAGGUAGUAGGUUGUAUAGUUuuagggucacacccaccacugggagauaacuauacaaucuacugucuuuccua | 6 to 27 |
| 8 | bta-let-7f | ugagguaguagauuguauaguu | ucagagUGAGGUAGUAGAUUGUAUAGUUgugggguagugauuuuacccuguucaggagauaacuauacaaucuauugccuucccuga | 7 to 29 |
| 9 | bta-miR-1 | uggaauguaaagaaguauguau | gcuugggaaacauacuucuuuaugugcccauauggaccugcuaagcuaUGGAAUGUAAAGAAGUAUGUAUuucagg | 49 to 70 |
| 10 | bta-miR-100 | aacccguagauccgaacuugug | ccuguugccacaAACCCGUAGAUCCGAACUUGUGgugguagugcacacaagcuugugucuauagguaugugucugu | 13 to 34 |
| 11 | bta-miR-101 | uacaguacugugauaacugaa | aggcugcccuggcucaguuaucacagugcugaugcuguccauucuaaaggUACAGUACUGUGAUAACUGAAGgauggcagcca | 51 to 71 |
| 12 | bta-miR-103 | agcagcauuguacagggcuauga | cugcccucggcuucuuuacagugcugccuuguugcauauggaucaAGCAGCAUUGUACAGGGCUAUGAaggc | 46 to 68 |
| 13 | bta-miR-106a | aaaagugcuuacagugcaggua | ccuuggccauguAAAAGUGCUUACAGUGCAGGUAgcuuuuugagaucuacugcaaugcaagcacuucuuacauuaccaugg | 13 to 34 |
| 14 | bta-miR-106b | uaaagugcugacagugcagau | ccugccggggcUAAAGUGCUGACAGUGCAGAUagugguccugugugcuaccgcacuguggguacuugcugcuccggcagg | 12 to 32 |
| 15 | bta-miR-107 | agcagcauuguacagggcuauc | cucucugcuuucagcuucuuuacaguguugccuuguggcauggaguucaAGCAGCAUUGUACAGGGCUAUCaaagcacaga | 50 to 71 |
| 16 | bta-miR-10a | uacccuguagauccgaauuugug | gaucugucugucuucuguauaUACCCUGUAGAUCCGAAUUUGUGuaaggaauuuugugaucacaaauucguaucuaggggaauauguaguugacauaaacacuccgcuc | 22 to 44 |
| 17 | bta-miR-10b | uacccuguagaaccgaauuugug | cagugacguugucuauauaUACCCUGUAGAACCGAAUUUGUGugguauccauguagucacagauucgauucuaggggaauauauggucgaugcaaaaac | 20 to 42 |
| 18 | bta-miR-1246 | aauggauuuuuggagcagg | caacauauuaAAUGGAUUUUUGGAGCAGGaaguuggaauagaggcuuucucagacaaauacuuuacugugcag | 11 to 29 |
| 19 | bta-miR-1248 | accuucuuguauaagcacugugcuaaa | uuuACCUUCUUGUAUAAGCACUGUGCUAAAauuacagaaaccaagauagccuugguuuuuguaauaaugcuagcagaguauuacacaagaagaagaggaacugca | 4 to 30 |
| 20 | bta-miR-1249 | acgcccuucccccccuucuuca | gggaggagggaggagaugggccacguucccucugccuggaACGCCCUUCCCCCCCUUCUUCAccu | 41 to 42 |
| 21 | bta-miR-125a | ucccugagacccuuuaaccugug | ugccggccucugcgUCCCUGAGACCCUUUAACCUGUGaggacguccagggucacaggugagguucuugggagccuggcguccggcc | 15 to 37 |
| 22 | bta-miR-125b | ucccugagacccuaacuuguga | cgcgcgccucucaaUCCCUGAGACCCUAACUUGUGAuguuuaccguuuaaauccacggguuaggcucuugggagcugcgagucgcgcc | 15 to 36 |
| 23 | bta-miR-1260b | aucccaccacugccacca | guugcugcugcucgccaucgAUCCCACCACUGCCACCAcugcugcuacugcuccgcaggugcugcugguggugaugaugauaguccggcgggggcgcugg | 21 to 38 |
| 24 | bta-miR-1271 | cuuggcaccuaguaaguacuca | cacccagaucagugCUUGGCACCUAGUAAGUACUCAguauauacuuguugagugccugcuaugugccaggcauugugcugagggcu | 15 to 36 |
| 25 | bta-miR-128 | ucacagugaaccggucucuuu | ugagcuguuggauucggggccguagcacugucugagagguuuacauuucUCACAGUGAACCGGUCUCUUUuucagcugcuuc | 50 to 70 |
| 26 | bta-miR-1296 | uuagggcccuggcuccaucucc | ccuaccuaacugggUUAGGGCCCUGGCUCCAUCUCCuuuaggaaaaccuucuguggggaguggggcuuugacccuaaccuagcugggcugu | 15 to 36 |
| 27 | bta-miR-1306 | ccaccuccccugcaaacgucc | CCACCUCCCCUGCAAACGUCCagugaugcagagguaauggacguuggcucugguggug | 1 to 21 |
| 28 | bta-miR-1307 | acucggcguggcgucggucgug | caucaagagucggcugagucacugccacugccuaccaaucucgaccggaccucgaccggcucgucuauauugccaaucgACUCGGCGUGGCGUCGGUCGUGguagauaggcggucauacagacgaguuuucaaguguugcucuggugac | 80 to 11 |
| 29 | bta-miR-130a | cagugcaauguuaaaagggcau | ugcugcgggccggagcucuuuucacauugugcuacugucugcgccugucacuagCAGUGCAAUGUUAAAAGGGCAUuggcugugcagcg | 55 to 76 |
| 30 | bta-miR-130b | cagugcaaugaugaaagggcau | ggccugccugacacucuuucccuguugcacuacugugcgccccuggcaagCAGUGCAAUGAUGAAAGGGCAUcggucaggcc | 51 to 72 |
| 31 | bta-miR-132 | uaacagucuacagccauggucg | ccgcccccgcgucuccagggcaaccguggcuuucgauuguuacugugggaaccggaggUAACAGUCUACAGCCAUGGUCGccccgcagcacgcccacgcgc | 59 to 80 |
| 32 | bta-miR-133a | uuugguccccuucaaccagcug | ugggaccgaaugcuuugcuaaagcugguaaaauggaaccaaaucaacuguucgauggaUUUGGUCCCCUUCAACCAGCUGuagcugcgcauugau | 59 to 80 |
| 33 | bta-miR-138 | agcugguguugugaaucaggccg | cuggcacggugcgguggggcAGCUGGUGUUGUGAAUCAGGCCGucgccaaucagagaacggcuacuucacaacaccagggucacaccccaccccagg | 21 to 43 |
| 34 | bta-miR-1388-5p | aggacuguccaaccugagaau | ccugggcggugccuucAGGACUGUCCAACCUGAGAAUggugagcauccagggacaaucucagguuugucagcccgcaaggugccguccccuc | 17 to 37 |
| 35 | bta-miR-139 | ucuacagugcacgugucuccagu | guguacUCUACAGUGCACGUGUCUCCAGUguggcucggaggcuggagacgcggcccuguuggaguaac | 7 to 29 |
| 36 | bta-miR-140 | uaccacaggguagaaccacgga | ucucucuguguccugccagugguuuuacccuaugguagguuacgucaugcuguucUACCACAGGGUAGAACCACGGAcaggauaccggggcacc | 56 to 77 |
| 37 | bta-miR-141 | uaacacugucugguaaagaugg | gaccggcucuggguccaucuuccagcacaguguuggauggucuaauggugaagcuccUAACACUGUCUGGUAAAGAUGGcccccggcugg | 58 to 79 |
| 38 | bta-miR-142-5p | cauaaaguagaaagcacuac | gacagugcagucaccCAUAAAGUAGAAAGCACUACuaacagcacuggaggguguaguguuuccuacuuuauggaugaguguacugug | 16 to 35 |
| 39 | bta-miR-1434-5p | guacaugaugacuaaaauuucu | caccuuGUACAUGAUGACUAAAAUUUCUucacuuugaccugauggcuauugaagaaaucuaaggucugaggug | 7 to 28 |
| 40 | bta-miR-145 | guccaguuuucccaggaaucccu | caccuuguccucacgGUCCAGUUUUCCCAGGAAUCCCUuagaugcuaagauggggauuccuggaaauacuguucuugaggucaugguu | 16 to 38 |
| 41 | bta-miR-1468 | cuccguuugccuguuuugcuga | guugaagaggugggugguuuCUCCGUUUGCCUGUUUUGCUGAuguucauuugacuuauucucagcaaaauaagcaaauggaaaauucauccaucaac | 21 to 42 |
| 42 | bta-miR-146a | ugagaacugaauuccauagguugu | cccauguguauccucagcuuUGAGAACUGAAUUCCAUAGGUUGUgucagugucagaccugugaaguuuaguucuuuagcugggauaucucuaucauccu | 21 to 44 |
| 43 | bta-miR-146b | ugagaacugaauuccauaggcugu | uaagagaacuuuggccaccuggcucUGAGAACUGAAUUCCAUAGGCUGUgagcucuagcaaaugcccuagggacucaguucuggugcccggcugugcuacaccauc | 26 to 49 |
| 44 | bta-miR-147 | gugugcggaaaugcuucugcua | uaugaaucuaguggaaacacuucugcacaggcuagauuauggauaccaGUGUGCGGAAAUGCUUCUGCUAcauuuuuagg | 49 to 70 |
| 45 | bta-miR-148a | ucagugcacuacagaacuuugu | gaggcaaaguucugagacacuccgacucugaauaugauagaagUCAGUGCACUACAGAACUUUGUcuc | 44 to 65 |
| 46 | bta-miR-148b | ucagugcaucacagaacuuugu | uuagcauuugaggugaaguucuguuauacacucaggcuguggcucucugaaagUCAGUGCAUCACAGAACUUUGUcucgaaagcuuucua | 54 to 75 |
| 47 | bta-miR-150 | ucucccaacccuuguaccagugu | ccucucuccucacggcccugUCUCCCAACCCUUGUACCAGUGUgugucucagacccugguacagguacggggaggcagggaccugggggaucccagcagc | 21 to 43 |
| 48 | bta-miR-151-5p | ucgaggagcucacagucuagu | ccugcccUCGAGGAGCUCACAGUCUAGUacgucucauccccuacuagacugaagcuccuugaggacagg | 8 to 28 |
| 49 | bta-miR-152 | ucagugcaugacagaacuuggg | uguccucccggcccagguucugugauacacuccgacucgggcucuggagcagUCAGUGCAUGACAGAACUUGGGcccggacggacc | 53 to 74 |
| 50 | bta-miR-153 | uugcauagucacaaaagugauc | cucacggcugccagcgucauuuuugugaucugcagcuaguauucucacuccagUUGCAUAGUCACAAAAGUGAUCacuggcagguguggc | 54 to 75 |
| 51 | bta-miR-154c | agauauugcacgguugaucucu | uucgaaaggagguuguccgugauguauuugcuuuauuuguggcAGAUAUUGCACGGUUGAUCUCUuuucuucauc | 44 to 65 |
| 52 | bta-miR-155 | uuaaugcuaaucgugauaggggu | UGUUAAUGCUAAUCGUGAUAGGGGUuuuuaccucggacugacuccuacauguuagcauuaaca | 3 to 25 |
| 53 | bta-miR-15a | uagcagcacauaaugguuugu | ccuuggaguaaagUAGCAGCACAUAAUGGUUUGUggauuuugaaaaggugcaggccauauugugcugccucaaaaauacaagg | 14 to 34 |
| 54 | bta-miR-15b | uagcagcacaucaugguuuaca | uugagaccuuaaaguacugUAGCAGCACAUCAUGGUUUACAuacuacagucaagaugcgaaucauuauuugcugcucuagaaauuuaaggaaauucau | 20 to 41 |
| 55 | bta-miR-16a | uagcagcacguaaauauuggug | gucagcagugccuUAGCAGCACGUAAAUAUUGGUGuuaagauucuaaaauuaucuccaguauuaacugugcugcugaaguaagguuggc | 14 to 35 |
| 56 | bta-miR-16b | uagcagcacguaaauauuggc | cauacuuguuccgcugUAGCAGCACGUAAAUAUUGGCguaguaaaauaaauauuaaacaccaauauuauugugcugcuuuagcgugacaggga | 17 to 37 |
| 57 | bta-miR-17-5p | caaagugcuuacagugcagguagu | gucagaauaauguCAAAGUGCUUACAGUGCAGGUAGUgauaugugcaucuacugcagugaaggcacuuguagcauuauggugac | 14 to 37 |
| 58 | bta-miR-181a | aacauucaacgcugucggugaguu | ugagcuccgagguugcuucagugAACAUUCAACGCUGUCGGUGAGUUuggaauuaaaaaucaaaaccaucgaccguugauuguacccuauggccaaccaccaucuccacc | 24 to 47 |
| 59 | bta-miR-181b | aacauucauugcugucgguggguu | cuugggcagagguucuuucuuaaaaggucacaaucAACAUUCAUUGCUGUCGGUGGGUUgaacuguguggacaagcucacugaacaaugagugcaacuguggccccgcau | 36 to 59 |
| 60 | bta-miR-181c | aacauucaaccugucggugaguuu | uugccaaggguuugggggAACAUUCAACCUGUCGGUGAGUUUgggcagcucaggcaaaccaucgaccguugaguggaccccgaggccuggaacugcc | 19 to 42 |
| 61 | bta-miR-1839 | aagguagauagaacaggucuuguu | aaugaaAAGGUAGAUAGAACAGGUCUUGUUugcaaaaugaauucaugaccuacauaucuaccaacagcaaug | 7 to 30 |
| 62 | bta-miR-185 | uggagagaaaggcaguuccuga | gggggugagggacUGGAGAGAAAGGCAGUUCCUGAugguccccuccccaggggcuggcuuuccuccggccccuccuucc | 14 to 35 |
| 63 | bta-miR-186 | caaagaauucuccuuuugggcu | ugcuuauaacuuucCAAAGAAUUCUCCUUUUGGGCUuucugauuuuauuuuaagcccaaaggugaauuuuuugggaaguuugagcu | 15 to 36 |
| 64 | bta-miR-188 | caucccuugcaugguggagggu | ugcucccucucucaCAUCCCUUGCAUGGUGGAGGGUgagcuuucugaaaaccccucccacaugcaggguuugcaggauggugagcc | 15 to 36 |
| 65 | bta-miR-18a | uaaggugcaucuagugcagaua | uguucUAAGGUGCAUCUAGUGCAGAUAgugaaguagauuagcaucuacugcccuaagugcuccuucuggca | 6 to 27 |
| 66 | bta-miR-18b | uaaggugcaucuagugcaguua | cuuguguUAAGGUGCAUCUAGUGCAGUUAgugaagcagcucagaaucuacugcccuaaaugcuccuucuggcaca | 8 to 29 |
| 67 | bta-miR-190b | ugauauguuugauauuggguu | ugcuucugugUGAUAUGUUUGAUAUUGGGUUguuuaauuaggaaccaacuaaaugucaaacauauucuuacagcaguag | 11 to 31 |
| 68 | bta-miR-191 | caacggaaucccaaaagcagcug | ggcuggacagcgggCAACGGAAUCCCAAAAGCAGCUGuugucuccagagcauuccagcugcgcuuggauuucguucccugcucuccugccu | 15 to 37 |
| 69 | bta-miR-192 | cugaccuaugaauugacagccag | agaccgagugcacagggcuCUGACCUAUGAAUUGACAGCCAGugcucuuguguccccucuggcugccaauuccauaggucacagguauguucgccucaaugccagc | 20 to 42 |
| 70 | bta-miR-193a-5p | ugggucuuugcgggcgagauga | ugggagcugagagcUGGGUCUUUGCGGGCGAGAUGAaggugucgguucaacuggccuacaaagucccaguccucggccccc | 15 to 36 |
| 71 | bta-miR-193b | aacuggcccacaaagucccgcuuu | guggucccagaaucgggguuuugagggcgagaugaguuuauguuuuauccAACUGGCCCACAAAGUCCCGCUUUuggggucau | 51 to 74 |
| 72 | bta-miR-194 | uguaacagcaacuccaugugga | uucuuaacggcgucaucgauUGUAACAGCAACUCCAUGUGGAcugugcgucaauuuccaguggagaugcuguuacuuuugauggcugccaauucacu | 21 to 42 |
| 73 | bta-miR-195 | uagcagcacagaaauauuggca | agcuccccuggcucUAGCAGCACAGAAAUAUUGGCAcugggaagaaagccugccaauauuggcugugcugcuccaggcaggguggug | 15 to 36 |
| 74 | bta-miR-196b | uagguaguuuccuguuguuggga | aacuggucggugauuUAGGUAGUUUCCUGUUGUUGGGAuccaccuuucucucgacagcacgacacugccuucauuacuucaguug | 16 to 38 |
| 75 | bta-miR-197 | uucaccaccuucuccacccagc | ggggcugugccggguagagagggcagugggagguaagagcucuucacccUUCACCACCUUCUCCACCCAGCagggccagca | 50 to 71 |
| 76 | bta-miR-19a | ugugcaaaucuaugcaaaacuga | gcaguccucuguuaguuuugcauaguugcacuacaagaagaauguaguUGUGCAAAUCUAUGCAAAACUGAugguggccugc | 49 to 71 |
| 77 | bta-miR-19b | ugugcaaauccaugcaaaacuga | cacuguucuaugguuaguuuugcagguuugcauccagcugugugauauucugcUGUGCAAAUCCAUGCAAAACUGAcugugguagug | 54 to 76 |
| 78 | bta-miR-200c | uaauacugccggguaaugaugga | cgucuuacccagcaguguuugggugcugguugggagucucUAAUACUGCCGGGUAAUGAUGGAgg | 41 to 63 |
| 79 | bta-miR-205 | uccuucauuccaccggagucug | cucuugUCCUUCAUUCCACCGGAGUCUGucucguacccaaccagauuucaguggagugaaguucaggag | 7 to 28 |
| 80 | bta-miR-20a | uaaagugcuuauagugcagguag | guagcacUAAAGUGCUUAUAGUGCAGGUAGuguuuaguuaucuacugcauuaugagcacuuaaaguacugc | 8 to 30 |
| 81 | bta-miR-20b | caaagugcucacagugcaggua | aguacCAAAGUGCUCACAGUGCAGGUAguuuuggcagcgcucuacuguagugugggcacuuccaguacu | 6 to 27 |
| 82 | bta-miR-210 | acugugcgugugacagcggcuga | ccuccaggcgcagggcagccacugcccaccgcacacugcgcugcuccggacccACUGUGCGUGUGACAGCGGCUGAucugucccugggcagcgcgacc | 54 to 76 |
| 83 | bta-miR-211 | uucccuuugucauccuuugcc | uccccuggcugugugaccugugggcUUCCCUUUGUCAUCCUUUGCCcaggguucugaguggggcagggacagcaaaggggugcucagucgucaccucccacagcauggag | 26 to 46 |
| 84 | bta-miR-215 | augaccuaugaauugacagaca | uguacaggaaaAUGACCUAUGAAUUGACAGACAacgugacuaagucugucugucauuucuguaggccaauguucuguau | 12 to 33 |
| 85 | bta-miR-21-5p | uagcuuaucagacugauguugacu | ugucgggUAGCUUAUCAGACUGAUGUUGACUguugaaucucauggcaacagcagucgaugggcugucugaca | 8 to 31 |
| 86 | bta-miR-221 | agcuacauugucugcuggguuu | ccaacauccaggucuagggcaugaaccuggcauacaauguagauuucuguguuuguugagcaacAGCUACAUUGUCUGCUGGGUUUcaggcuaccuggaaacacguucuu | 65 to 86 |
| 87 | bta-miR-222 | agcuacaucuggcuacugggu | gcugcuggaauguguagguacccucaauggcucaguagccaguguagauccugucuuuuguaaucaguAGCUACAUCUGGCUACUGGGUcucugauggcaucuucuaccu | 69 to 89 |
| 88 | bta-miR-223 | ugucaguuugucaaauacccca | cccagccuccugcagugccaugcuccguguauuugacaagcugaguuggacacuccauguaguagUGUCAGUUUGUCAAAUACCCCAaguguggcauaugccuagcag | 66 to 87 |
| 89 | bta-miR-22-5p | aguucuucaguggcaagcuuua | ggcugagccgcaguAGUUCUUCAGUGGCAAGCUUUAuguccugacccagcuaaagcugccaguugaagaacuguugcccucugcc | 15 to 36 |
| 90 | bta-miR-2284j | gaaaaguucguucagguuuu | guuggugggccGAAAAGUUCGUUCAGGUUUUccaaguuacagaauaacccagacaaacuguuuggccagcccaau | 12 to 31 |
| 91 | bta-miR-2284w | aagaguuuguucggguuucuc | uuucaauuauuggguuggccAAGAGUUUGUUCGGGUUUCUCcauaagcuguauagaaaaaccucaaugaacucuuuggccaacccaauauuuugcu | 21 to 41 |
| 92 | bta-miR-2284x | ugaaaaguucguucggguuuu | ggauuuaguauuggguuggcUGAAAAGUUCGUUCGGGUUUUuccauaagaacucaaauaaacuuuuuggccaacccaguaauaacacugaugcacc | 21 to 41 |
| 93 | bta-miR-2285aa | aaaacuggaacgaacuuuugggc | caaaaaguucguccagauuuuuccauaagauguuacagaAAAACUGGAACGAACUUUUGGGC | 40 to 62 |
| 94 | bta-miR-2285c | aaaccugaacaaacuuuuuggc | gccgaaauguucguugaaguuuuuccauggggugucacggaagAAACCUGAACAAACUUUUUGGC | 44 to 65 |
| 95 | bta-miR-2285e | aaaccugaacgaacuuuuuggc | gccaaaaaguucguucggauuucucuguaagcugguguggauAAACCUGAACGAACUUUUUGGC | 43 to 64 |
| 96 | bta-miR-2285f | aaaaccugaaugaacuuuuugg | uuggccaaaaaguucauuugaguuuuccugcacuaucuuauggaAAAACCUGAAUGAACUUUUUGGguaa | 45 to 66 |
| 97 | bta-miR-2285j | aaaaaccagaacgaacuuuuug | auuggucaaaaauuuguuuggguuuuuccaguugauguugAAAAACCAGAACGAACUUUUUGgccaacc | 41 to 62 |
| 98 | bta-miR-2285k | aaaaccggaaugaacuuuuug | augggcaaaaaguuugcuuggauuuuucugugagauauuaugggAAAACCGGAAUGAACUUUUUGacuga | 45 to 65 |
| 99 | bta-miR-2285n | aaaaacccgaaugaacuuuugg | cacuggguuagccaaaaaguucgucccgguguuuccauaagauguaacggAAAAACCCGAAUGAACUUUUGGccaacccagu | 51 to 72 |
| 100 | bta-miR-2285o | aaacccgaacgaacuuuugg | auaggauuggccaaagaguucauuuggguuuuucuagugagcuguaaacaaaAAACCCGAACGAACUUUUGGcugacucaau | 53 to 72 |
| 101 | bta-miR-2285p | aaaaacuugagugaacuuuugg | acuggguuggccaaauuguucauuuggguuuuuccguaacaucuuauggAAAAACUUGAGUGAACUUUUGGgucagcccag | 50 to 71 |
| 102 | bta-miR-2285u | gaaaaacccgaacgaacuuu | ggaguaugcuggcguuggccaaaauguucauuccaauuuuucuguaccaucuuacaGAAAAACCCGAACGAACUUUuuggcuaacccaauaguaaauaccc | 57 to 76 |
| 103 | bta-miR-2285x | gaaaaaucugaaugaacuuuugg | uauauuggguuggccaaaaaguuuguucaggguuuuucuaagauguuacgGAAAAAUCUGAAUGAACUUUUGGccaaccuaauaua | 51 to 73 |
| 104 | bta-miR-2285y | aaaacccaaacaaacuuuuuu | uguugaguuggccaaaaaguucguuuggauuucucuguaauaucuuacggaAAAACCCAAACAAACUUUUUUgaacaacccaaua | 52 to 72 |
| 105 | bta-miR-2312 | aaaaccugaacgaacuuuuc | uugggcuggccaaacaguuugucuggguuauucuguaacuuggAAAACCUGAACGAACUUUUCggcccaccaa | 44 to 63 |
| 106 | bta-miR-2313-5p | agugcagcugaggaccaaggca | gggcuggAGUGCAGCUGAGGACCAAGGCAgggcugcaugcauucacaugccaguuccacgcugcaugccggccu | 8 to 29 |
| 107 | bta-miR-2317 | cucugugaugacaauaccugaua | aguuCUCUGUGAUGACAAUACCUGAUAgagguacugauaccucuaucauaccugauaccugauagagauacu | 5 to 27 |
| 108 | bta-miR-2318 | guguaugaugaauuaucugacc | ugucaggaaccccGUGUAUGAUGAAUUAUCUGACCcggccguugccucucauguaacgcuucucauggcucucacugca | 14 to 35 |
| 109 | bta-miR-2331-5p | ggcuuccgugccugcagaugucu | ggguGGCUUCCGUGCCUGCAGAUGUCUgugaauuccucaaggcugagacccugcagccaaagaagcuaccc | 5 to 27 |
| 110 | bta-miR-2335 | agauaaugaugacuaacugaau | guAGAUAAUGAUGACUAACUGAAUugaugacuuugggcaugguuuaguaaucaguuucaugauuucuau | 3 to 24 |
| 111 | bta-miR-2340 | ggacuucccugguggucuugug | acaacuccaaggauacgaggcaagguaagaucagagugaugagaGGACUUCCCUGGUGGUCUUGUGcagggugugu | 45 to 66 |
| 112 | bta-miR-2383 | cugagugaugacugcugacc | ggacggugagcuaagguggggucaugggccaaggccaguggcugagCUGAGUGAUGACUGCUGACCuacaccgucu | 47 to 66 |
| 113 | bta-miR-23a | aucacauugccagggauuucca | ggccggcugggguuccuggggaugggauuugcugccugucacaaAUCACAUUGCCAGGGAUUUCCAaccgacc | 45 to 66 |
| 114 | bta-miR-24 | gugccuacugagcugauaucagu | cuccgGUGCCUACUGAGCUGAUAUCAGUucucauuuuacacacuggcucaguucagcaggaacaggag | 6 to 28 |
| 115 | bta-miR-2419-5p | aucgcaucaacacucgucuguu | ccucuaugAUCGCAUCAACACUCGUCUGUUcuuaaauaacauugagaacaggcgggugcuaauacgaucauagagg | 9 to 30 |
| 116 | bta-miR-2435 | aaacucgaaugaacucuuuggc | ggaAAACUCGAAUGAACUCUUUGGCugacucaguauuauguuuagcaucagagagucaucagcauguucc | 4 to 25 |
| 117 | bta-miR-24-3p | uggcucaguucagcaggaacag | cucugccucccgugccuacugagcugaaacacaguugauuugugcacacUGGCUCAGUUCAGCAGGAACAGg | 50 to 71 |
| 118 | bta-miR-2440 | ugcagugaugagacccugga | uccagcugugguggggacuguuuggugcgggguggggccagcugUGCAGUGAUGAGACCCUGGAaugagcgcuggg | 45 to 64 |
| 119 | bta-miR-2468 | auaggaacauggaagauuguca | gauuggcAUAGGAACAUGGAAGAUUGUCAgucaucaucuauuucugccaauuuuccauguuccugugccaguc | 8 to 29 |
| 120 | bta-miR-2478 | guaucccacuucugacacca | guugggggucgguggugagcaagcagagacuacauagguugccuuGUAUCCCACUUCUGACACCAuguauugucaac | 46 to 65 |
| 121 | bta-miR-2484 | gagcuaugaugacuuugauugcau | uuaucugaaaauguaugcaggguuauuauaauuaaaaagguGAGCUAUGAUGACUUUGAUUGCAUugaucacauga | 42 to 65 |
| 122 | bta-miR-25 | cauugcacuugucucggucuga | ggccaguguugagaggcggagacuugggcaauugcuggacgcugccccgggCAUUGCACUUGUCUCGGUCUGAcagugccggcc | 52 to 73 |
| 123 | bta-miR-26a | uucaaguaauccaggauaggcu | aaggccguggccucgUUCAAGUAAUCCAGGAUAGGCUgugcaggucccaaggggccuauucucgguuacuugcacgcggacgcgggccug | 16 to 37 |
| 124 | bta-miR-26b | uucaaguaauucaggauagguu | ugcccgggacccagUUCAAGUAAUUCAGGAUAGGUUgugugcuguccagccuguucuccauuacuuggcucgggggccggugccc | 15 to 36 |
| 125 | bta-miR-27a-5p | agggcuuagcugcuugugagca | uggccuggggagcAGGGCUUAGCUGCUUGUGAGCAgguccacaucaaaucguguucacaguggcuaaguuccgccccc | 14 to 35 |
| 126 | bta-miR-27b | uucacaguggcuaaguucugc | accucucugacgaggugcagagcuuagcugauuggugaacagugacugguuuccgcuuugUUCACAGUGGCUAAGUUCUGCaccugaagagaaggug | 61 to 81 |
| 127 | bta-miR-28 | aaggagcucacagucuauugag | gguccuugcccucAAGGAGCUCACAGUCUAUUGAGuugccuuucugacuuucccacuagauugagagcuccuggagggcaggcacu | 14 to 35 |
| 128 | bta-miR-2887 | cgggaccgggguccggugcg | cguCGGGACCGGGGUCCGGUGCGgagagcccuucgucccgggacacggggcgcggcc | 4 to 23 |
| 129 | bta-miR-2903 | uuccgcgcucuacgccagc | cgccgcuccgcucccggUUCCGCGCUCUACGCCAGCccgcugccugccgggcuggugcaggccgcggagccgggcggcccggcaggca | 18 to 36 |
| 130 | bta-miR-2904 | gggagccucgguuggccuc | acggcagcgccgcGGGAGCCUCGGUUGGCCUCggauagccggucccccgccguccccgccggcgggccgu | 14 to 32 |
| 131 | bta-miR-296-5p | gagggccccccccaauccu | aggacccuuccgGAGGGCCCCCCCCAAUCCUguugugcuugguucagaggguugggcggaggcuuuccugaagggucu | 13 to 31 |
| 132 | bta-miR-29b | uagcaccauuugaaaucaguguu | cuucaggaagcugguuucauauggugguuuagauuuaaauagugauugucUAGCACCAUUUGAAAUCAGUGUUcuuggggg | 51 to 73 |
| 133 | bta-miR-29c | uagcaccauuugaaaucgguua | aucucuuacacaggcugaccgauuucuccugguguucagagucuguuuuugucUAGCACCAUUUGAAAUCGGUUAugauguaggggga | 54 to 75 |
| 134 | bta-miR-29d-5p | ugaccgauuucuccugguguu | aucucuuacacaggcUGACCGAUUUCUCCUGGUGUUcagagucuguuuuugucuagcaccauuugaaaucgauuaugauguaggggga | 16 to 36 |
| 135 | bta-miR-301a | cagugcaauaguauugucaaagcau | acugcuaacgaaugcucugacuuuauugcacuacuguacuuuacagcuagCAGUGCAAUAGUAUUGUCAAAGCAUcugaaagcag | 51 to 75 |
| 136 | bta-miR-301b | cagugcaaugauauugucaaagcau | uccugcuggccgcaggugcucugacgagguugcacuacugugcuuugaggagCAGUGCAAUGAUAUUGUCAAAGCAUccgggaccagccuuggag | 53 to 77 |
| 137 | bta-miR-30a-5p | uguaaacauccucgacuggaagcu | cUGUAAACAUCCUCGACUGGAAGCUgugaggcugcagaaaggcuuucagucggauguuugcagcugc | 2 to 25 |
| 138 | bta-miR-30b-5p | uguaaacauccuacacucagcu | ccaaguuuucaguucaUGUAAACAUCCUACACUCAGCUguaacacacgagucggcugggagguggauguuuacuucagcugacuugga | 17 to 38 |
| 139 | bta-miR-30c | uguaaacauccuacacucucagc | cagacuguaaccaugccguagugugUGUAAACAUCCUACACUCUCAGCugugagcucgagguggcugggagaggguuguuuacuccuucugccauggaaaacguc | 26 to 48 |
| 140 | bta-miR-30d | uguaaacauccccgacuggaagcu | guuguUGUAAACAUCCCCGACUGGAAGCUguaccacacagcuaagcuuucagucagauguuugcugcuac | 6 to 29 |
| 141 | bta-miR-30e-5p | uguaaacauccuugacuggaagcu | gggcagucuuugcuacUGUAAACAUCCUUGACUGGAAGCUguaaggcguugcaaggagcuuucagucggauguuuacagcggcaggcugcca | 17 to 40 |
| 142 | bta-miR-30f | uguaaacacccuacacucucagcu | gagggccagauacUGUAAACACCCUACACUCUCAGCUgugcacagugagaaagcugggagaaggcuguuuacucucucugccuu | 14 to 37 |
| 143 | bta-miR-31 | aggcaagaugcuggcauagcu | uccuguaacuuggaacuggagaggAGGCAAGAUGCUGGCAUAGCUguugaacugcgaaccugcuaugccaacauauugccaucucucuuguccg | 25 to 45 |
| 144 | bta-miR-3141 | gagggcggguggaggagg | ccgcccccgucuccaccccgccccccggagccgcugaguggcggcagcggccccaggaccggguuuccucagacaaGAGGGCGGGUGGAGGAGGaagcgg | 77 to 36 |
| 145 | bta-miR-32 | uauugcacaugacuaaguugcau | ggaggUAUUGCACAUGACUAAGUUGCAUguugucacggccucagugcaauuuaguaugugugauauuuuc | 6 to 28 |
| 146 | bta-miR-320a | aaaagcuggguugagagggcga | cccgcggcgucucgcuccccuccgccuucucuucccgguucuucccggagucgggAAAAGCUGGGUUGAGAGGGCGAaaaag | 56 to 77 |
| 147 | bta-miR-324 | cgcauccccuagggcauuggugu | aacuggcuaugccucccCGCAUCCCCUAGGGCAUUGGUGUaaagcuggagacccacugccccaggugcugcuggggguuguagucugac | 18 to 40 |
| 148 | bta-miR-326 | ccucugggcccuuccuccag | cucgucugucuguugggcuggaggcagggccuuugugaaggcggguugugcucagaucgCCUCUGGGCCCUUCCUCCAGcccagaggcggauuca | 60 to 79 |
| 149 | bta-miR-328 | cuggcccucucugcccuuccgu | cuguuucggagccuggagcgggggggcaggaggggcucagggagauagugugugcugcccCUGGCCCUCUCUGCCCUUCCGUccccuguucc | 61 to 82 |
| 150 | bta-miR-330 | gcaaagcacacggccugcagaga | cuucggcgaucacugccucucugggccugugucuuaggcucugcaagaucaaccgaGCAAAGCACACGGCCUGCAGAGAggcagcgcucagcuc | 57 to 79 |
| 151 | bta-miR-331-5p | ucuagguauggucccagg | gaguuugguuuuguuuggguuuguUCUAGGUAUGGUCCCAGGgaucccagaucaaaccaggccccugggccuauccuagaaccaaccuaa | 25 to 42 |
| 152 | bta-miR-335 | ucaagagcaauaacgaaaaaugu | uuuugggcgggggUCAAGAGCAAUAACGAAAAAUGUuugucauaaaccguuuuucauuauugcuccugaccuccucucauuugcuguacuca | 14 to 36 |
| 153 | bta-miR-338 | uccagcaucagugauuuuguuga | gcacgggccguccuccccaacaauauccuggugcugagugaugacacacgcaacUCCAGCAUCAGUGAUUUUGUUGAagagggcagcugcca | 55 to 77 |
| 154 | bta-miR-339a | ucccuguccuccaggagcucac | ggggcagccgcugUCCCUGUCCUCCAGGAGCUCACuugguccggccgugcgcuccucgaggccagagcccgugucugc | 14 to 35 |
| 155 | bta-miR-33a | gugcauuguaguugcauugca | cugcgGUGCAUUGUAGUUGCAUUGCAuguucuggcgguacccgugcaauguuuccacagugcaucacag | 6 to 26 |
| 156 | bta-miR-340 | uccgucucaguuacuuuauagcc | uuguaccuggugugauuauaaagcaaugagacugauugucaugugucguuugugggaUCCGUCUCAGUUACUUUAUAGCCauaccugguaucuua | 58 to 80 |
| 157 | bta-miR-342 | ucucacacagaaaucgcacccaucu | uggaagcgggugcgaggcgaggggugcuaucugugguugaggacacggcaaaugaaacugUCUCACACAGAAAUCGCACCCAUCUccucggccc | 61 to 85 |
| 158 | bta-miR-345-5p | gcugacuccuaguccagugcu | acccaaacccaggucuGCUGACUCCUAGUCCAGUGCUugugauggcuggugggcccugaacuaggggucuggaggccuggguuugaauauc | 17 to 37 |
| 159 | bta-miR-34a | uggcagugucuuagcugguugu | ggccagcugugaguguuucuuUGGCAGUGUCUUAGCUGGUUGUugugaguaauaaugcaggaagcaaucagcaaguauacugcccuagaagugcugcacguuguggg | 22 to 43 |
| 160 | bta-miR-34b | aggcaguguaauuagcugauug | gugcucgguuuguAGGCAGUGUAAUUAGCUGAUUGuacucucaugcuuacaaucacuaguuccacugccaucaaaacaaggcac | 14 to 35 |
| 161 | bta-miR-34c | aggcaguguaguuagcugauug | agucuaguuacuAGGCAGUGUAGUUAGCUGAUUGcuaauaauaccaaucacuaaccacacggccagguaaaaagauu | 13 to 34 |
| 162 | bta-miR-3601 | ucucgugacaugaugauccccga | cccagaccuuucaguuaucaaucugucacaagugcacagugguaUCUCGUGACAUGAUGAUCCCCGAgaugucugagg | 45 to 67 |
| 163 | bta-miR-361 | uuaucagaaucuccagggguac | ggagcUUAUCAGAAUCUCCAGGGGUACuuauaauuugaaaaagucccccaggugugauucugauuugcuuc | 6 to 27 |
| 164 | bta-miR-362-5p | aauccuuggaaccuaggugugagu | cucgAAUCCUUGGAACCUAGGUGUGAGUgcuguucuagugcaacacaccuauucaaggauucaaa | 5 to 28 |
| 165 | bta-miR-363 | auugcacgguauccaucugcg | uguugucggguggaucacgaugcaauuuugauuaguauaauaggagaaaaAUUGCACGGUAUCCAUCUGCGaac | 51 to 71 |
| 166 | bta-miR-365-3p | uaaugccccuaaaaauccuuau | agagugcucgaggacagcaagaaaaaugagggacuuucaggggcagcuguguuuucugacucagucaUAAUGCCCCUAAAAAUCCUUAUuguucuugcagugugcaucagg | 68 to 89 |
| 167 | bta-miR-374a | uuauaauacaaccugauaagug | uacaucggccaUUAUAAUACAACCUGAUAAGUGuuacagcacuuaucagguuguauuguaauugucugugua | 12 to 33 |
| 168 | bta-miR-374b | auauaauacaaccugcuaagug | gaagaaauccuacucggauggAUAUAAUACAACCUGCUAAGUGuccuagcacuuaucagguuguauuaucauuguccgugucuauggcucucguc | 22 to 43 |
| 169 | bta-miR-375 | uuuuguucguucggcucgcguga | ccccgcgacgagccccucgcacaaaccggaccugagcgUUUUGUUCGUUCGGCUCGCGUGAggc | 39 to 61 |
| 170 | bta-miR-376d | aucauagaggaaaauccacau | gguauuuaaaagguagauuuuccuucuaugauuacggguuuggugauuaAUCAUAGAGGAAAAUCCACAUuuucgguaucaa | 50 to 70 |
| 171 | bta-miR-376e | aacauagaggaaaauccacauu | ugauauucaaaagguggauauuccuucuauguuuacaggauugacagcuaAACAUAGAGGAAAAUCCACAUUuuaaguaucu | 51 to 72 |
| 172 | bta-miR-378 | acuggacuuggagucagaaggc | agggcuccugacuccagguccuguguguuaccucgaaauagcACUGGACUUGGAGUCAGAAGGCcu | 43 to 64 |
| 173 | bta-miR-378c | acuggacuuggagucagaagu | caaaaccaccACUGGACUUGGAGUCAGAAGUuccaugugugagucuugguagggccaguggauugcuaa | 11 to 31 |
| 174 | bta-miR-409a | agguuacccgagcaacuuugcau | ugguacgcggggagAGGUUACCCGAGCAACUUUGCAUcuggacgacgaauguugcucggugaaccccuuuucgguauca | 15 to 37 |
| 175 | bta-miR-411a | auaguagaccguauagcguacg | ugguacuuggagagAUAGUAGACCGUAUAGCGUACGcuuuaucugugacguauguaacacgguccacuaacccucaguauca | 15 to 36 |
| 176 | bta-miR-421 | aucaacagacauuaauugggcgc | gcacauuguaggccucauuaaauguuuguugaaugaaaaaaugaaucAUCAACAGACAUUAAUUGGGCGCcugcucugugaucuc | 48 to 70 |
| 177 | bta-miR-423-5p | ugaggggcagagagcgagacuuu | auaaaggaaguuaggcUGAGGGGCAGAGAGCGAGACUUUucuauuuuccaaaagcucggucugaggccccucagucuugcuuccuaccccgcgc | 17 to 39 |
| 178 | bta-miR-425-5p | augacacgaucacucccguuga | gaaagcgcuuuggaAUGACACGAUCACUCCCGUUGAgugggcacccaagaagccaucgggaaugucguguccgcccagugcucuuuc | 15 to 36 |
| 179 | bta-miR-4286 | accccacuccugguacc | ggccuacACCCCACUCCUGGUACCagcugcuugggcccccccuguggcacaugccucugagauggagggauuaaug | 8 to 24 |
| 180 | bta-miR-449a | uggcaguguauuguuagcuggu | ugugugauggguUGGCAGUGUAUUGUUAGCUGGUugaauaugugagugccaucagcuaacaugcaacugcuaucuuauugcauguaua | 13 to 34 |
| 181 | bta-miR-451 | aaaccguuaccauuacugaguuu | cuugggguggcgaggAAACCGUUACCAUUACUGAGUUUaguaaugguaacgguucucuugcugcacccaga | 16 to 38 |
| 182 | bta-miR-454 | uagugcaauauugcuuauagggu | cuguuuauuaccagauccuagaacccuaucgauauugucucugcuguguaaauagcucugagUAGUGCAAUAUUGCUUAUAGGGUuuugguguuugggaagaacaaugggcaug | 63 to 85 |
| 183 | bta-miR-484 | ucaggcucaguccccucccgau | gUCAGGCUCAGUCCCCUCCCGAUaaaccucuaaauagggaccuucccggggggcuaccucggc | 2 to 23 |
| 184 | bta-miR-485 | agaggcuggccgugaugaauucg | acuugaagAGAGGCUGGCCGUGAUGAAUUCGauucaucaaagcgagucauacacggcucuccucucuuuuagu | 9 to 31 |
| 185 | bta-miR-486 | uccuguacugagcugccccgag | gccagcuuggaccugcguccucccugacgggUCCUGUACUGAGCUGCCCCGAGgcccuucgcugugcccagcucgggucagcucaguaccgggcgcgucggggugggagucggccggaagcagg | 32 to 53 |
| 186 | bta-miR-487b | aaucguacagggucauccacuu | uugguacuuggagagugguuaucccuguccuguucguuuuacucaugucgAAUCGUACAGGGUCAUCCACUUuuucaguaucaa | 51 to 72 |
| 187 | bta-miR-491 | aguggggaacccuuccaugagg | uugacuuagcuggguAGUGGGGAACCCUUCCAUGAGGaguagaacacuccuuaugcaagauucccuucuaccugacuggguugg | 16 to 37 |
| 188 | bta-miR-494 | ugaaacauacacgggaaaccuc | ucgauacuugaaggagagguuauccguguugucuucucuuuauuuaugaUGAAACAUACACGGGAAACCUCuuuuuuaguaucaa | 50 to 71 |
| 189 | bta-miR-497 | cagcagcacacugugguuugua | ccaccccaguccugcucccgcccCAGCAGCACACUGUGGUUUGUAcggcacuguggccacguccaaaccacacugugguguuagagcgagggugggggaggcaccgcugagg | 24 to 45 |
| 190 | bta-miR-499 | uuaagacuugcagugauguuu | gggcgggcggccgUUAAGACUUGCAGUGAUGUUUaacuccucuccacgugaacaucacagcaagucugugcugcuucccguccccacgcugccugggcagggu | 14 to 34 |
| 191 | bta-miR-500 | uaauccuugcuaccugggugaga | gcucccccucucUAAUCCUUGCUACCUGGGUGAGAgugcuuucugaaugcaaugcaccugggcaaggauucugagagagggagc | 13 to 35 |
| 192 | bta-miR-502a | aaugcaccugggcaaggauuca | ugcccaccccucuaauccuugcucucugggugcuagugcugucuccaugcAAUGCACCUGGGCAAGGAUUCAgagagggugggcu | 51 to 72 |
| 193 | bta-miR-503-5p | uagcagcgggaacaguacug | agccgugcccUAGCAGCGGGAACAGUACUGcagugggcaauuggugaucuggaguauuguuucugcugcccgggcaagacugg | 11 to 30 |
| 194 | bta-miR-505 | cgucaacacuugcugguuuccu | aaauugaugcacccagugggggagccaggaaguauugauguuucuaccaguuuagCGUCAACACUUGCUGGUUUCCUcucuggagcguca | 56 to 77 |
| 195 | bta-miR-545-5p | ucaguaaauguuuauuggaug | cccagccuggcacauucguaggccUCAGUAAAUGUUUAUUGGAUGaauaaaugaauggcucaucaacaaacauuuauugugugccugcuaacgugaucuccacagg | 25 to 45 |
| 196 | bta-miR-574 | ugagugugugugugugagugugug | uucuuuugagcuuacuucccagucucgaauccgugUGAGUGUGUGUGUGUGAGUGUGUGugagagug | 36 to 59 |
| 197 | bta-miR-6119-5p | agagguaaaaaauugauuugacu | auuuugAGAGGUAAAAAAUUGAUUUGACUaguucuuuaacacaucuagcaaaucauuuuuuacucuccaaaaagaac | 7 to 29 |
| 198 | bta-miR-6123 | ugccaagcccacguucaaagg | agccugcUGCCAAGCCCACGUUCAAAGGcuguuucuucaaaguuaauggugccgccuuugagcugggaagggaagcgggcag | 8 to 28 |
| 199 | bta-miR-628 | augcugacauauuuacuagagg | auagcuguugugucacuuccucAUGCUGACAUAUUUACUAGAGGguaaaauuaauaaccuucuaguaagaguggcagucgaagggaagggcucau | 23 to 44 |
| 200 | bta-miR-6517 | ucaggguccgugagcuccucggc | ccgccgguucUCAGGGUCCGUGAGCUCCUCGGCguacucggagaacauucgccggaacuccggguccuggaaggcgga | 11 to 33 |
| 201 | bta-miR-652 | aauggcgccacuaggguugug | acgaauggcuaugcacugcacaacccuaggagagggugccauucacauagacuagaauugAAUGGCGCCACUAGGGUUGUGcagugcacaaccuacac | 61 to 81 |
| 202 | bta-miR-6524 | uuacucugaguaaccuaacug | aacaaggacaccaguuaggccacucagaguaacuaucaguaacuaacuggUUACUCUGAGUAACCUAACUGguauccuugu | 51 to 71 |
| 203 | bta-miR-6531 | uagacuugguguacuuugagaga | uaccuacuucUAGACUUGGUGUACUUUGAGAGAcaguguuguuuuuugcuccuuuuuuauacuugcuaugucuggacguggcaa | 11 to 33 |
| 204 | bta-miR-660 | uacccauugcauaucggagcug | cugcuccuucucccgUACCCAUUGCAUAUCGGAGCUGugaauucucaaagcaccuccuaugugcauggauuacaggaggg | 16 to 37 |
| 205 | bta-miR-664b | uauucauuuaucucccagccuac | caggcuaggagaaaugauuggauagaaaauuuuauucUAUUCAUUUAUCUCCCAGCCUAC | 38 to 60 |
| 206 | bta-miR-671 | aggaagcccuggaggggcuggag | gccgccgaccuggcaggccaggaagagGAGGAAGCCCUGGAGGGGCUGGAGgugauggauguuuuccuccgguucucagggcuccaccuuuuccgggccguggagccagggcuggugc | 29 to 51 |
| 207 | bta-miR-677 | cucacugaugagcagcuucugac | CUCACUGAUGAGCAGCUUCUGACuuucguucuucugaguuugcugaagccagaugccauuucugag | 1 to 23 |
| 208 | bta-miR-7 | uggaagacuagugauuuuguuguu | guggagcagccagccccaucUGGAAGACUAGUGAUUUUGUUGUUgucuccugcgcucaacaacaagucccagucugccgcauggugcuggccaccgca | 21 to 44 |
| 209 | bta-miR-744 | ugcggggcuagggcuaacagca | gguugggcggggUGCGGGGCUAGGGCUAACAGCAggcucacugacgguuucccggaaaccacgcacaugcuguugccacuaaccucaaccuuacucgguc | 13 to 34 |
| 210 | bta-miR-769 | ugagaccuccggguucugagcu | gacuuggugcugauucccggcgcucugaccUGAGACCUCCGGGUUCUGAGCUgugauguugcuucccagcugggaucucuggggucucgguucagggucgggaccucuggguucugagc | 31 to 52 |
| 211 | bta-miR-7859 | aaaaacuggcagcuucauguaa | augugaaauuaccaguauuugucugcuuugaccuacAAAAACUGGCAGCUUCAUGUAA | 37 to 58 |
| 212 | bta-miR-92a | uauugcacuugucccggccugu | cuuucuacacagguugggaucgguugcaaugcuguguuucuguauggUAUUGCACUUGUCCCGGCCUGUugaguuugg | 48 to 69 |
| 213 | bta-miR-92b | uauugcacucgucccggccucc | cgggccccgggcgggcgggagggacgggacgcggugcaguguuguucuuuccccugccaaUAUUGCACUCGUCCCGGCCUCCggcccccucggccc | 61 to 82 |
| 214 | bta-miR-93 | caaagugcuguucgugcaggua | cugggggcucCAAAGUGCUGUUCGUGCAGGUAgugugaucaccugaccuacugcugagccagcacuucccgagcccc | 11 to 32 |
| 215 | bta-miR-95 | uucaacggguauuuauugagca | aacacagcgggcgcucaauaaauguuuguugaauugagaugcgcuaaaUUCAACGGGUAUUUAUUGAGCAcccacucugug |  |
| 216 | bta-miR-9-5p | ucuuugguuaucuagcuguaug | ggaggcccguuucucUCUUUGGUUAUCUAGCUGUAUGagugccacagagccgucauaaagcuagauaaccgaaaguagaaaugacucuca | 16 to 37 |
| 217 | bta-miR-98 | ugagguaguaaguuguauuguu | aggacucugcucaugcuggggUGAGGUAGUAAGUUGUAUUGUUgugggguagggauuuuaggccccaauuugaagauaacuauacaacuuacuacuuucccugguguguagcacauuca | 22 to 43 |
| 218 | bta-miR-99a-5p | aacccguagauccgaucuugu | cccauuggcauaAACCCGUAGAUCCGAUCUUGUggugaaguggaccgcacaagcucgcuucuaugggucugugucagugug | 13 to 33 |
| 219 | bta-miR-99b | cacccguagaaccgaccuugcg | ggcaccCACCCGUAGAACCGACCUUGCGgggccuucgccgcacacaagcucgugucuguggguccguguc | 7 to 28 |
| 220 | bta-miR-136 | acuccauuuguuuugaugaugga | uuggaugagcccucggaggACUCCAUUUGUUUUGAUGAUGGAuucuuacgcuccaucaucgucucaaaugagucuucagaggguuccaucau | 20 to 42 |
| 221 | bta-miR-184 | uggacggagaacugauaagggu | ccagucacauccccuuaucacuuuuccagccagcuuugugacucuaacuguUGGACGGAGAACUGAUAAGGGUaggugauuga | 52 to 73 |
| 222 | bta-miR-190a | ugauauguuugauauauuaggu | ugcaggccucugugUGAUAUGUUUGAUAUAUUAGGUuguuauuuaauccaacuauauaucaaacauauuccuacagugucuugcc | 15 to 36 |
| 223 | bta-miR-299 | ugguuuaccgucccacauacau | aagaaaUGGUUUACCGUCCCACAUACAUucugaauauguaugugggacgguaaaccgcuucuu | 7 to 28 |
| 224 | bta-miR-378b | acuugacuuggagucagaaggc | cuggaccaccagggaaauccugauuuuguuucuuauuaaggggagguucaguauagagcaaacagcACUUGACUUGGAGUCAGAAGGCuuagguccaa | 67 to 88 |
| 225 | bta-miR-381 | uauacaagggcaagcucucugu | uacucagagcgagguugcccuuuguauauucgguuuuuugauguagaaUAUACAAGGGCAAGCUCUCUGUgagua | 49 to 70 |
| 226 | bta-miR-424-5p | cagcagcaauucauguuuuga | ucguugacuccgaggggaugCAGCAGCAAUUCAUGUUUUGAagugcuuuaaacgguucaaaacgugaggcgcugcuauacccccuugcgaggaagu | 21 to 41 |
| 227 | bta-miR-496 | ugaguauuacauggccaaucuc | ccgagucggguacucgaauggagguuguccaugguguguucauuuuauuuaugaUGAGUAUUACAUGGCCAAUCUCcuuucaguacuaaauucuucauggg | 55 to 76 |
| 228 | bta-miR-532 | caugccuugaguguaggaccgu | gacuugcuuucucucuuaCAUGCCUUGAGUGUAGGACCGUuggcaucuuaauuacccucccacacccaaggcuugcaggagagcca | 19 to 40 |
| 229 | bta-miR-877 | guagaggagauggcgcaggg | gcucgagaagGUAGAGGAGAUGGCGCAGGGgacacgggcuaagacugggggcucccgggaccccccaacauguguccucuucucccuccucccagguguacg | 11 to 30 |
| 230 | bta-miR-2427 | aggucauuucaaagagggcug | caauauuaucAGGUCAUUUCAAAGAGGGCUGaugggaccaaauccaggggcucuuaucacugugaccaaagauug | 11 to 31 |
| 231 | bta-miR-143 | ugagaugaagcacuguagcucg | gcguccugucucccagccugaggugcagugcugcaucucuggucaguugggagucUGAGAUGAAGCACUGUAGCUCGggaagggagaaguuguucugcagc | 56 to 77 |
| 232 | bta-miR-206 | uggaauguaaggaagugugugg | ugcuucccaaggccacaugcuucuuuauauccccauacggauuacuuugcuaUGGAAUGUAAGGAAGUGUGUGGuuucggcgagcg | 53 to 74 |
| 233 | bta-miR-2284b | aaaaguucguuugguuuuuuc | uuggcugAAAAGUUCGUUUGGUUUUUUCcacaagauguuagagaaagaaccugauugaacuuuuuggccaa | 8 to 28 |
| 234 | bta-miR-2284y | aaaaguucguucggguuuuuc | aauaaccccuguuaaggccugcagaacuugcuugaucuauuggguuggccaAAAAGUUCGUUCGGGUUUUUCcuuagucccaaacuaaguuuuuggccaacccaauacaaucuguugggcuucc | 52 to 72 |
| 235 | bta-miR-2285ad | aaaacccaaaugaacuuuuugg | aaaaguucguuucaguuuuuccauaacauuuuacagAAAACCCAAAUGAACUUUUUGG | 37 to 58 |
| 236 | bta-miR-2285z | ccagaaaguucauucagguccu | uaauuuaaaauguguuggCCAGAAAGUUCAUUCAGGUCCUucccuaacaucuuacagaaaaccugaaugaacuuuuuggccaacaacauuuaauug | 19 to 40 |
| 237 | bta-miR-29e | uagcaucauuugaaaucaguguuu | cuucuggaagcugguuucacaugguggcuuagauuuuuccaucuuuguaucUAGCAUCAUUUGAAAUCAGUGUUUuaggag | 52 to 75 |
| 238 | bta-miR-33b | gugcauugcuguugcauugc | gcgggcggccccgcgGUGCAUUGCUGUUGCAUUGCaugugugaggcaggugcagugccucggcagugcagcccggagccggccccuggcaccgc | 16 to 35 |
| 239 | bta-miR-3431 | ccucagucagccuuguggaugu | auacccaaugaaugcgaaaaCCUCAGUCAGCCUUGUGGAUGUauguucugcagaccugacaucuagaggacugacugaaauuuucacuuucagcuaa | 21 to 42 |
| 240 | bta-miR-6516 | uuugcaguaacaggugugaac | UUUGCAGUAACAGGUGUGAACauucuagcagcaguuugaugaucauguaugauacugcaaac | 1 to 21 |
| 241 | bta-miR-6520 | uugaguauugucagagagagc | gucccucuccUUGAGUAUUGUCAGAGAGAGCgauucugugcccacccugagucgucucuccgccuguacucagccagcagcgcc | 11 to 31 |
| 242 | bta-let-7a-3p | cuauacaaucuacugucuuuc | gggugagguaguagguuguauaguuuggggcucugcccugcuaugggauaaCUAUACAAUCUACUGUCUUUCcu | 52 to 72 |
| 243 | bta-miR-129-3p | aagcccuuaccccaaaaagcau | cugcccuucgcgaaucuuuuugcggucugggcuugcuguacauaacucaauagccggAAGCCCUUACCCCAAAAAGCAUucgcggagggcgcac | 58 to 79 |
| 244 | bta-miR-1343-3p | cuccuggggcccgcacucuc | ggcuucggugcuggggagcggcccccgggcgggccucugcucuggcccCUCCUGGGGCCCGCACUCUCgcuccgggcc | 49 to 68 |
| 245 | bta-miR-1388-3p | aucucagguuugucagcccgca | ccugggcggugccuucaggacuguccaaccugagaauggugagcauccagggacaAUCUCAGGUUUGUCAGCCCGCAaggugccguccccuc | 56 to 77 |
| 246 | bta-miR-142-3p | aguguuuccuacuuuauggaug | gacagugcagucacccauaaaguagaaagcacuacuaacagcacuggaggguguAGUGUUUCCUACUUUAUGGAUGaguguacugug | 55 to 76 |
| 247 | bta-miR-151-3p | cuagacugaagcuccuugagg | ccugcccucgaggagcucacagucuaguacgucucauccccuaCUAGACUGAAGCUCCUUGAGGacagg | 44 to 64 |
| 248 | bta-miR-17-3p | acugcagugaaggcacuugu | gucagaauaaugucaaagugcuuacagugcagguagugauaugugcaucuACUGCAGUGAAGGCACUUGUagcauuauggugac | 51 to 70 |
| 249 | bta-miR-193a-3p | aacuggccuacaaagucccagu | ugggagcugagagcugggucuuugcgggcgagaugaaggugucgguucAACUGGCCUACAAAGUCCCAGUccucggccccc | 49 to 70 |
| 250 | bta-miR-21-3p | aacagcagucgaugggcugucu | ugucggguagcuuaucagacugauguugacuguugaaucucauggcAACAGCAGUCGAUGGGCUGUCUgaca | 47 to 68 |
| 251 | bta-miR-22-3p | aagcugccaguugaagaacug | ggcugagccgcaguaguucuucaguggcaagcuuuauguccugacccagcuaAAGCUGCCAGUUGAAGAACUGuugcccucugcc | 53 to 73 |
| 252 | bta-miR-2284t-3p | aaacucgaaugaauguuuuggc | guuggccgaaacauucacucggguuuuuccauagcaucuuaacagaaAAACUCGAAUGAAUGUUUUGGCcaac | 48 to 69 |
| 253 | bta-miR-2331-3p | acccugcagccaaagaagcua | ggguggcuuccgugccugcagaugucugugaauuccucaaggcugagACCCUGCAGCCAAAGAAGCUAccc | 48 to 68 |
| 254 | bta-miR-2355-3p | auuguccuugcuguuuggagau | gucauucccagauacaauggacaauaugcuguuauaauuguguggcAUUGUCCUUGCUGUUUGGAGAUaauacugcugac | 47 to 68 |
| 255 | bta-miR-23b-3p | aucacauugccagggauuaccac | ggguuccuggcaugcugauuugugacuuaagauuaaaAUCACAUUGCCAGGGAUUACCAC | 38 to 60 |
| 256 | bta-miR-2411-3p | gcugaacugucuuacucccacaucc | gaggaaauguggagugacugucagaugcagccagcagaauaagugguuugGCUGAACUGUCUUACUCCCACAUCCuc | 51 to 75 |
| 257 | bta-miR-2419-3p | caggcgggugcuaauacgauca | ccucuaugaucgcaucaacacucgucuguucuuaaauaacauugagaaCAGGCGGGUGCUAAUACGAUCAuagagg | 49 to 70 |
| 258 | bta-miR-27a-3p | uucacaguggcuaaguuccg | uggccuggggagcagggcuuagcugcuugugagcagguccacaucaaaucguGUUCACAGUGGCUAAGUUCCGccccc | 54 to 73 |
| 259 | bta-miR-296-3p | gaggguugggcggaggcuuucc | aggacccuuccggagggccccccccaauccuguugugcuugguucaGAGGGUUGGGCGGAGGCUUUCCugaagggucu | 47 to 68 |
| 260 | bta-miR-29d-3p | uagcaccauuugaaaucgauua | aucucuuacacaggcugaccgauuucuccugguguucagagucuguuuuugucUAGCACCAUUUGAAAUCGAUUAugauguaggggga | 54 to 75 |
| 261 | bta-miR-331-3p | gccccugggccuauccuagaa | gaguuugguuuuguuuggguuuguucuagguauggucccagggaucccagaucaaaccagGCCCCUGGGCCUAUCCUAGAAccaaccuaa | 61 to 81 |
| 262 | bta-miR-345-3p | ccugaacuaggggucuggag | acccaaacccaggucugcugacuccuaguccagugcuugugauggcuggugggcCCUGAACUAGGGGUCUGGAGgccuggguuugaauauc | 55 to 74 |
| 263 | bta-miR-362-3p | aacacaccuauucaaggauuc | cucgaauccuuggaaccuaggugugagugcuguucuagugcAACACACCUAUUCAAGGAUUCaaa | 42 to 62 |
| 264 | bta-miR-369-3p | aauaauacaugguugaucuuu | ugaagggagaucgaccguguuauauucgcuuuauugacuucgAAUAAUACAUGGUUGAUCUUUucucag | 43 to 63 |
| 265 | bta-miR-423-3p | aagcucggucugaggccccucagu | auaaaggaaguuaggcugaggggcagagagcgagacuuuucuauuuuccaaAAGCUCGGUCUGAGGCCCCUCAGUcuugcuuccuaccccgcgc | 52 to 75 |
| 266 | bta-miR-425-3p | aucgggaaugucguguccgccc | gaaagcgcuuuggaaugacacgaucacucccguugagugggcacccaagaagccAUCGGGAAUGUCGUGUCCGCCCagugcucuuuc | 55 to76 |
| 267 | bta-miR-455-3p | gcaguccaugggcauauacacu | ucccuggcgugaggguaugugccuuuggacuacaucguggaagccagcaccauGCAGUCCAUGGGCAUAUACACUugccucaaggccua | 54 to 75 |
| 268 | bta-miR-503-3p | ggaguauuguuucugcugcccgg | agccgugcccuagcagcgggaacaguacugcagugggcaauuggugaucuGGAGUAUUGUUUCUGCUGCCCGGgcaagacugg | 51 to 73 |
| 269 | bta-miR-545-3p | aucaacaaacauuuauugugug | cccagccuggcacauucguaggccucaguaaauguuuauuggaugaauaaaugaauggcucAUCAACAAACAUUUAUUGUGUGccugcuaacgugaucuccacagg | 62 to 83 |
| 270 | bta-miR-6119-3p | gcaaaucauuuuuuacucuccaa | auuuugagagguaaaaaauugauuugacuaguucuuuaacacaucuaGCAAAUCAUUUUUUACUCUCCAAaaagaac | 48 to 70 |
| 271 | bta-miR-760-3p | cggcucugggucugugggga | ggaggaugcugcagcguggggcgcgucgccccccucaguccaccagagcccggauaccuuagaaauuCGGCUCUGGGUCUGUGGGGAGcgaaaugcaacccaaacuccauuuugccg | 68 to 87 |
| 272 | bta-miR-99a-3p | caagcucgcuucuaugggu | cccauuggcauaaacccguagauccgaucuuguggugaaguggaccgcaCAAGCUCGCUUCUAUGGGUcugugucagugug | 50 to 68 |
| 273 | bta-miR-126-3p | cguaccgugaguaauaaugcg | ugacgggacauuauuacuuuugguacgcgcugugacacuucaaacuCGUACCGUGAGUAAUAAUGCGcuguca | 47 to 67 |
| 274 | bta-miR-424-3p | caaaacgugaggcgcugcuau | ucguugacuccgaggggaugcagcagcaauucauguuuugaagugcuuuaaacgguuCAAAACGUGAGGCGCUGCUAUacccccuugcgaggaagu | 58 to 78 |
