# Supplementary material for: MicroRNA expression profiling in PBMCs of Indian water Buffalo (Bubalus bubalis) infected with Brucella and Johne’s disease
Source: ExRNA. 2020 May 22;2(1):8. doi: 10.1186/s41544-020-00049-y (PMC7242893; doi:10.1186/s41544-020-00049-y)
Supplement: Supplementary file 2 — Additional file 2. [file 41544_2020_49_MOESM2_ESM.doc]

**Supplementary file 2. List of the novel mature star miRNAs (no bovine-homologue is available in miRBase ) identified in the bubaline PBMCs from diseased vis-à-vis healthy control animals**

| **SN** | **Novel miRNA** | **Mature* miRNA sequence (5’-3’)** | **Pre-miRNA sequence (5’-3’)** | **Position** |
| --- | --- | --- | --- | --- |
| 1 | bta-let-7d-3p | cuauacgaccugcugccuuucu | ccuaggaagagguaguagguugcauaguuuucgggcagggauuuugcccacaaggagguaaCUAUACGACCUGCUGCCUUUCUuagg | 62 to 83 |
| 2 | bta-let-7i-3p | cugcgcaagcuacugccuugcu | cuggcugagguaguaguuugugcuguuggucggguugugacauugcccgcuguggagauaaCUGCGCAAGCUACUGCCUUGCUa | 62 to 83 |
| 3 | bta-mir-132-5p | accguggcuuucgauuguuacu | ccgcccccgcgucuccagggcaACCGUGGCUUUCGAUUGUUACUgugggaaccggagguaacagucuacagccauggucgccccgcagcacgcccacgcgc | 23 to 44 |
| 4 | bta-mir-1388-5p | aggacuguccaaccugagaaug | ccugggcggugccuucAGGACUGUCCAACCUGAGAAUGgugagcauccagggacaaucucagguuugucagcccgcaaggugccguccccuc | 17 to 38 |
| 5 | bta-mir-140-5p | cagugguuuuacccuaugguagg | ucucucuguguccugcCAGUGGUUUUACCCUAUGGUAGGuuacgucaugcuguucuaccacaggguagaaccacggacaggauaccggggcacc | 17 to 39 |
| 6 | bta-mir-142-5p | cccauaaaguagaaagcacuac | gacagugcagucaCCCAUAAAGUAGAAAGCACUACuaacagcacuggaggguguaguguuuccuacuuuauggaugaguguacugug | 14 to 35 |
| 7 | bta-mir-148b-5p | gaaguucuguuauacacucaggcu | uuagcauuugagguGAAGUUCUGUUAUACACUCAGGCUguggcucucugaaagucagugcaucacagaacuuugucucgaaagcuuucua | 15 to 38 |
| 8 | bta-mir-15a-3p | aggccauauugugcugccuca | ccuuggaguaaaguagcagcacauaaugguuuguggauuuugaaaaggugcAGGCCAUAUUGUGCUGCCUCAaaaauacaagg | 52 to 72 |
| 9 | bta-mir-15b-3p | cgaaucauuauuugcugcucua | uugagaccuuaaaguacuguagcagcacaucaugguuuacauacuacagucaagaugCGAAUCAUUAUUUGCUGCUCUAgaaauuuaaggaaauucau | 58 to 79 |
| 10 | bta-mir-185-3p | aggggcuggcuuuccuccggc | gggggugagggacuggagagaaaggcaguuccugaugguccccuccccAGGGGCUGGCUUUCCUCCGGCcccuccuucc | 49 to 69 |
| 11 | bta-mir-191-3p | gcugcgcuuggauuucguuccc | ggcuggacagcgggcaacggaaucccaaaagcagcuguugucuccagagcauuccaGCUGCGCUUGGAUUUCGUUCCCugcucuccugccu | 57 to 78 |
| 12 | bta-mir-19b-5p | aguuuugcagguuugcauccagc | cacuguucuaugguuAGUUUUGCAGGUUUGCAUCCAGCugugugauauucugcugugcaaauccaugcaaaacugacugugguagug | 16 to 38 |
| 13 | bta-mir-20a-3p | acugcauuaugagcacuuaaagu | guagcacuaaagugcuuauagugcagguaguguuuaguuaucuACUGCAUUAUGAGCACUUAAAGUacugc | 44 to 66 |
| 14 | bta-mir-21-3p | caacagcagucgaugggcuguc | ugucggguagcuuaucagacugauguugacuguugaaucucauggCAACAGCAGUCGAUGGGCUGUCugaca | 46 to 67 |
| 15 | bta-mir-210-5p | agccacugcccaccgcacacugc | ccuccaggcgcagggcAGCCACUGCCCACCGCACACUGCgcugcuccggacccacugugcgugugacagcggcugaucugucccugggcagcgcgacc | 17 to 39 |
| 16 | bta-mir-22-3p | aagcugccaguugaagaacugu | ggcugagccgcaguaguucuucaguggcaagcuuuauguccugacccagcuaAAGCUGCCAGUUGAAGAACUGUugcccucugcc | 53 to 74 |
| 17 | bta-mir-2468-3p | ccaauuuuccauguuccugugc | gauuggcauaggaacauggaagauugucagucaucaucuauuucugCCAAUUUUCCAUGUUCCUGUGCcaguc | 47 to 68 |
| 18 | bta-mir-25-5p | aggcggagacuugggcaauugcu | ggccaguguugagAGGCGGAGACUUGGGCAAUUGCUggacgcugccccgggcauugcacuugucucggucugacagugccggcc | 14 to 36 |
| 19 | bta-mir-26b-3p | ccuguucuccauuacuuggcu | ugcccgggacccaguucaaguaauucaggauagguugugugcuguccagCCUGUUCUCCAUUACUUGGCUcgggggccggugccc | 50 to 70 |
| 20 | bta-mir-27b-5p | agagcuuagcugauuggugaaca | accucucugacgaggugcAGAGCUUAGCUGAUUGGUGAACAgugacugguuuccgcuuuguucacaguggcuaaguucugcaccugaagagaaggug | 19 to 41 |
| 21 | bta-mir-29a-5p | acugauuucuuuugguguucagag | augACUGAUUUCUUUUGGUGUUCAGAGucaauauaauuuucuagcaccaucugaaaucgguuau | 4 to 27 |
| 22 | bta-mir-29b-1-5p | gcugguuucauauggugguuuaga | cuucaggaaGCUGGUUUCAUAUGGUGGUUUAGAuuuaaauagugauugucuagcaccauuugaaaucaguguucuuggggg | 10 to 33 |
| 23 | bta-mir-29b-2-5p | gcugguuucacaugguggcuuaga | cuucuggaaGCUGGUUUCACAUGGUGGCUUAGAuuuuuccaucuuuguaucuagcaccauuugaaaucaguguuuuaggag | 10 to 33 |
| 24 | bta-mir-29c-5p | accgauuucuccugguguucag | aucucuuacacaggcugACCGAUUUCUCCUGGUGUUCAGagucuguuuuugucuagcaccauuugaaaucgguuaugauguaggggga | 18 to 39 |
| 25 | bta-mir-30b-3p | cugggagguggauguuuacuuc | ccaaguuuucaguucauguaaacauccuacacucagcuguaacacacgagucggCUGGGAGGUGGAUGUUUACUUCagcugacuugga | 55 to 76 |
| 26 | bta-mir-30e-3p | cuuucagucggauguuuacagc | gggcagucuuugcuacuguaaacauccuugacuggaagcuguaaggcguugcaaggagCUUUCAGUCGGAUGUUUACAGCggcaggcugcca | 59 to 80 |
| 27 | bta-mir-324-3p | ccacugccccaggugcugcugg | aacuggcuaugccuccccgcauccccuagggcauugguguaaagcuggagacCCACUGCCCCAGGUGCUGCUGGggguuguagucugac | 53 to 74 |
| 28 | bta-mir-339a-3p | gcgcuccucgaggccagagcc | ggggcagccgcugucccuguccuccaggagcucacuugguccggccguGCGCUCCUCGAGGCCAGAGCCcgugucugc | 49 to 69 |
| 29 | bta-mir-33a-3p | caauguuuccacagugcauca | cugcggugcauuguaguugcauugcauguucuggcgguacccgugCAAUGUUUCCACAGUGCAUCAcag | 46 to 66 |
| 30 | bta-mir-340-5p | uuauaaagcaaugagacugauu | uuguaccuggugugaUUAUAAAGCAAUGAGACUGAUUgucaugugucguuugugggauccgucucaguuacuuuauagccauaccugguaucuua | 16 to 37 |
| 31 | bta-mir-361-3p | cccccaggugugauucugauuugc | ggagcuuaucagaaucuccagggguacuuauaauuugaaaaaguCCCCCAGGUGUGAUUCUGAUUUGCuuc | 45 to 68 |
| 32 | bta-mir-362-3p | aacacaccuauucaaggauuca | cucgaauccuuggaaccuaggugugagugcuguucuagugcAACACACCUAUUCAAGGAUUCAaa | 42 to 63 |
| 33 | bta-mir-425-3p | aucgggaaugucguguccgcc | gaaagcgcuuuggaaugacacgaucacucccguugagugggcacccaagaagccAUCGGGAAUGUCGUGUCCGCCcagugcucuuuc | 55 to 75 |
| 34 | bta-mir-6119-3p | caaaucauuuuuuacucuccaa | auuuugagagguaaaaaauugauuugacuaguucuuuaacacaucuagCAAAUCAUUUUUUACUCUCCAAaaagaac | 49 to 70 |
| 35 | bta-mir-7-2-3p | caacaaaucacagucugccaua | uggauguuggucuaguucuguguggaagacuagugauuuuguuguuuuuagauaacugaaucgaCAACAAAUCACAGUCUGCCAUAuggcacaggccaugccucuacag | 65 to 86 |
| 36 | bta-mir-92a-1-5p | agguugggaucgguugcaaugcu | cuuucuacacAGGUUGGGAUCGGUUGCAAUGCUguguuucuguaugguauugcacuugucccggccuguugaguuugg | 11 to 33 |
